# Comparative pathology of experimental pulmonary tuberculosis in animal models

**DOI:** 10.3389/fvets.2023.1264833

**Published:** 2023-10-12

**Authors:** Laura Hunter, Inés Ruedas-Torres, Irene Agulló-Ros, Emma Rayner, Francisco J. Salguero

**Affiliations:** ^1^Pathology Department, UK Health Security Agency (UKHSA), Porton Down, Salisbury, United Kingdom; ^2^School of Biosciences and Medicine, University of Surrey, Guildford, United Kingdom; ^3^Department of Anatomy and Comparative Pathology and Toxicology, UIC Zoonosis y Enfermedades Emergentes ENZOEM, University of Córdoba, International Excellence Agrifood Campus, Córdoba, Spain

**Keywords:** tuberculosis, animal models, pathology, granuloma, mycobacteria, lung

## Abstract

Research in human tuberculosis (TB) is limited by the availability of human tissues from patients, which is often altered by therapy and treatment. Thus, the use of animal models is a key tool in increasing our understanding of the pathogenesis, disease progression and preclinical evaluation of new therapies and vaccines. The granuloma is the hallmark lesion of pulmonary tuberculosis, regardless of the species or animal model used. Although animal models may not fully replicate all the histopathological characteristics observed in natural, human TB disease, each one brings its own attributes which enable researchers to answer specific questions regarding TB immunopathogenesis. This review delves into the pulmonary pathology induced by *Mycobacterium tuberculosis* complex (MTBC) bacteria in different animal models (non-human primates, rodents, guinea pigs, rabbits, cattle, goats, and others) and compares how they relate to the pulmonary disease described in humans. Although the described models have demonstrated some histopathological features in common with human pulmonary TB, these data should be considered carefully in the context of this disease. Further research is necessary to establish the most appropriate model for the study of TB, and to carry out a standard characterisation and score of pulmonary lesions.

## 1. Introduction

Tuberculosis (TB) is the second leading infectious disease killer in humans after COVID-19 (SARS-2) (1). *Mycobacterium tuberculosis* (Mtb) is part of the *Mycobacterium tuberculosis* complex (MTBC), which consists of nine closely related species (*M. tuberculosis, M. bovis, M. africanum, M. canetti, M. microti, M. mungi, M. orygis*, *M. caprae, M. pinnipedii and M. suricattae*) that can cause TB in both humans and animals.

Although TB has been studied for many decades, there are still many aspects of the immunopathogenesis which are not fully understood. The only licenced vaccine, the Bacillus Calmette-Guérin (BCG), has variable efficacy dependant on various factors including the target population and the age at vaccination. Therefore, there is still much research ongoing to find new vaccines and therapeutics.

Research into human TB is limited by the availability of infected human tissues due to the challenges associated with obtaining serial lung biopsies throughout the progression of the disease (2–4). Furthermore, pathogenic characteristics and severity is inherently altered by therapy and treatment, which is a major barrier (2, 3).

Whilst humans are the natural host for Mtb, infection and subsequent disease is observed in many animal species, making these good models in which to study the disease progression in a way that is prohibited in humans (3, 4). No animal model of TB fully replicates all the pathological characteristics observed in human TB disease progression; therefore, the vast majority of data is generated using many different animal models and must be considered carefully when relating to human disease (2, 3). Mycobacteria within the MTBC are also important pathogens for animal health, and animal models provide a useful tool for bovine TB (bTB) research (5–8).

Regardless of the species, the lung granuloma is the hallmark of pulmonary tuberculosis (9). Its first description was made by Anton Ghon, an Austrian pathologist, who made significant contributions to the understanding of primary TB and its anatomical pathology in humans (10, 11). It is known that Mtb infects alveolar macrophages, which release multiple cytokines to recruit additional macrophages, dendritic cells, and lymphocytes, leading to the formation of an early granuloma (9). This event triggers the recruitment of various types of immune cells, including neutrophils, which can produce chemokines and cytokines in response to the infection, attracting additional immune cells to the site of infection (9, 12). Infected macrophages play an important role during granuloma formation, serving as a central platform around where the rest of the immune cells are located, and giving rise to the typical, spherical morphology (9, 12). With the progression of the infection, mature macrophages undergo a morphological change, referred to as ‘epithelioid differentiation’ due to its capacity to form tight, interdigitated, cellular junctions, also known as epithelioid histiocytes (12–14). Macrophages can also fuse together to form multinucleated giant cells (MNGCs, also known as Langhans giant cells) or differentiate into foamy macrophages, characterised by the accumulation of lipids within their cytoplasm (9, 12–14).

This review paper will focus on the histopathological features of pulmonary granulomas in different animal models of experimental and natural MTBC infection (Table 1) and how they relate to the disease progression observed in humans.

**Table 1:** Main histopathological features in pulmonary granulomas in the different TB models.

	Necrosis	Mineralisation	Fibrosis	MNGCs	AFB	Cavitary lesions	References and comments
Human	**+**	**+**	**+**	**+**	**+**	**+**	(8, 10, 13–30). Cavitary lesions are common (2, 17, 24). Liquefaction (necrosis) present (24).
Mouse	**−/+**	**−/+**	**−/+**	**−**	**+**	**−/+**	(8, 12, 19, 20, 31–43). Absence of central necrosis in common strains (8, 12, 19, 20, 31). MNGCs not present, only occasional (16, 19, 32). Fibrosis seen in C5BL/6 mice (19). Foamy macrophages essential in mice (19, 32). Central necrosis seen in C3HeB/FeJ and HIS-NSG mice (35, 37–39). Development of cavitary lesions in C3HeB/FeJ mice strain (37). Abundant AFBs
Rat	**−/+**	**−/+**	**−/+**	**+**	**+**	**−**	Absence of central necrosis in common rats (40, 41) F344/N-rnu rat strain develops central non-caseous necrosis (40) MNGCs are present (40, 41, 43). Cotton rats develop caseous necrosis and calcification (42)
Guinea Pig	**+**	**+**	**−/+**	**+**	**+**	**−**	(44–53). Fibrosis is occasional (45). Mineralisation is a key feature (47). Associated lymphadenopathy and lymphangitis are important (51).
NHP	**+**	**+**	**+**	**+**	**+**	**+**	(29, 33, 35, 36, 54–71). Cavitation is rare in Old World Monkeys but seen in New World Monkeys such as the common marmoset (36).
Rabbit	**+**	**+**	**−/+**	**+**	**+**	**+**	(25, 72–75, 88) Development of liquefaction is present (72–75). Good model for TB meningitis (75). Mineralisation and evidence of scant AFBs (91). Fibrosis is present (25, 88).
Zebrafish	**+**	**−**	**+**	**+**	**+**	**−**	(66, 92, 94, 95, 97, 101) Absence of mineralisation (76–78). Fewer lymphocytes than in other models (76–78).
Cattle	**+**	**+**	**+**	**+**	**+**	**−**	(6, 67, 93, 96, 98, 102) AFB abundant in more developed necrotic lesions (5, 66, 92) MNGCs and foamy macrophages are abundant (94)
Goats	**+**	**+**	**+**	**+**	**+**	**+**	(79, 80, 103, 105, 106). Liquefactive necrosis is observed (79, 80, 103). Cavitary lesions are present (79, 80, 103).

Key: +, presence of feature, −, absence of feature, +/−, differing results. MNGCs – Multinucleated giant cells. AFB – Acid fast bacilli.

## 2. Tuberculosis in humans

**Figure 1:**
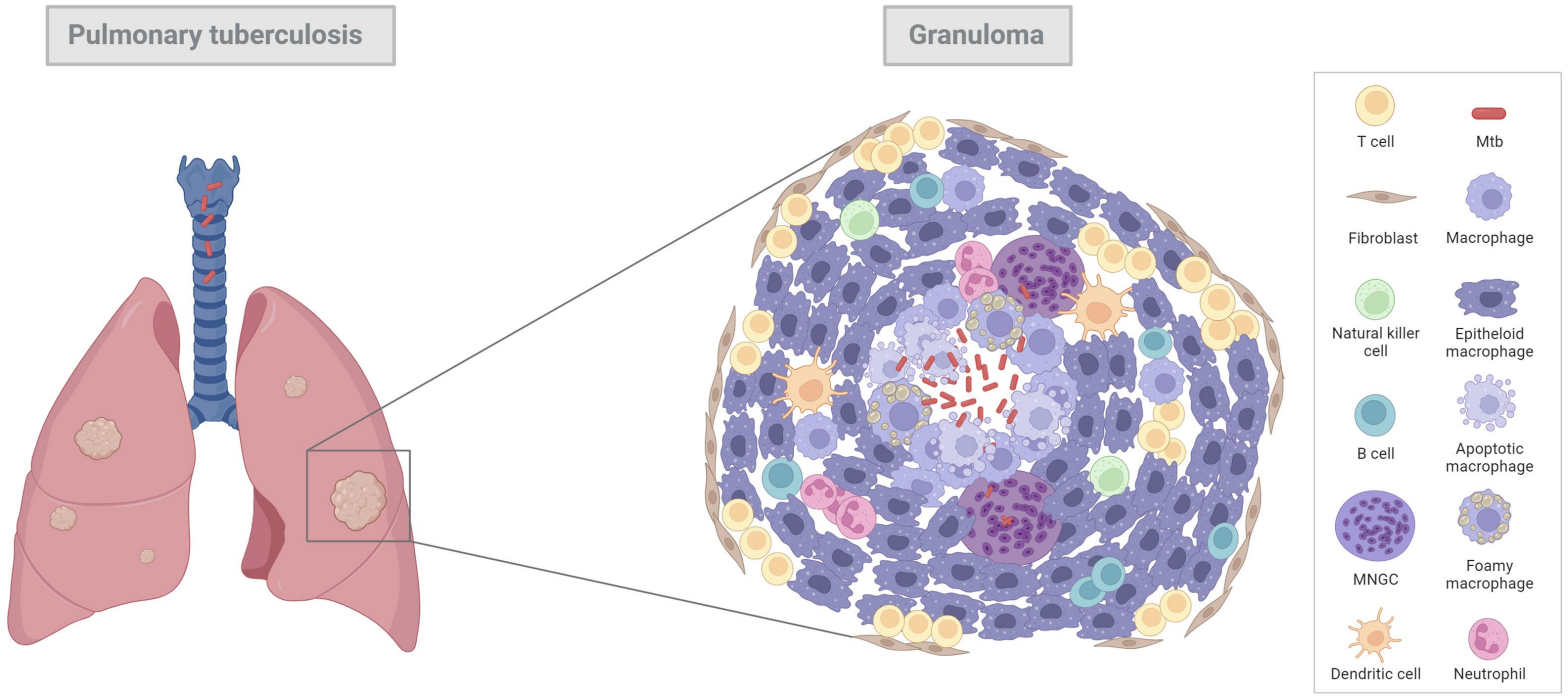
Diagram of a general pulmonary tuberculous granuloma. Mtb infects alveolar macrophages which release cytokines to attract additional macrophages, dendritic cells and lymphocytes, leading to an early granuloma. With the progression of the infection, tuberculous granulomas become bigger, more structured, and organised lesions, characterised by a central area of necrosis (infected macrophages) that harbours the mycobacteria. Surrounding this necrotic core there are mature macrophages, called “epithelioid macrophages” (due to its capacity to from interdigital junctions), multinucleated giant cells (MNGCs) and other immune cell types such as lymphocytes, neutrophils, natural killer cells, and dendritic cells to a less extent. In the outer layer there are large number of T cells and fibroblasts which try to encapsule the lesion. Image created with BioRender.com.

Early pulmonary granulomas induced by Mtb in human lungs are characterised by a central region of large epithelioid cells (CD68+ cells by immunohistochemistry [IHC]), surrounded by a mixture of macrophages and predominantly CD4+ T cells, with a smaller number of CD8+ T cells and MNGCs (15). The advanced model of human tuberculous granulomas describes well-structured and organised lesions, characterised by a central area of necrosis that harbours the mycobacteria (9, 11, 14–17) (Figure 1; Table 1). This necrosis is the result of the death of infected macrophages that form the central region of the granuloma and receives the name of caseum due to its ‘cheese-like’ consistency observed grossly (9, 12, 18). The necrotic core provides nutritional support and leads the mycobacteria to survive for decades in a latent state (9, 11). The initial layer surrounding the necrotic centre is populated with T lymphocytes (mainly CD4+ T cells), macrophages and MNGCs (CD68+ cells), diffusely distributed (9, 11, 14, 17, 19). These MNGCs are an important feature of human TB in lung tissue (17). In the outer layers there are large numbers of CD8+ T cells (15). Some authors also describe the presence of prominent, B cell aggregates which are located more distant to the necrotic core, and which are situated in a background of T lymphocytes and mycobacteria-containing macrophages diffusely distributed (15, 17). Whilst this leukocyte infiltration contributes to the local immune response, it can also lead to significant damage in the affected lung (11). As the disease progresses, the necrotic core can calcify (dystrophic mineralisation) and be encapsulated by a fibrotic rim (12, 16). Calcification is usually demonstrated with Von Kossa staining and fibrosis can be visualised with trichome stains such as Martius Scarlett Blue (MSB) or Masson’s Trichrome (16). It is thought that the main function of the granuloma is to prevent the mycobacterial spread, but it could also serve as a physical barrier to prevent the penetration of anti-TB drugs (14).

Zieh-Neelsen (ZN) staining is the routine technique to detect the presence of acid-fast bacilli (AFB) in tissues (15, 16, 19–21). However, IHC and immunofluorescence (IF) are also applied to identify AFB in tissue specimens (11, 22–24).

During human disease where TB reactivation has occurred, it is typical to observe the formation of thin-walled cavities (2, 18). These derive from the replacement of large areas of necrosis in primary granulomas with an open space that often contains a high number of neutrophils and necrotic cellular debris (2). The wall of the cavity is composed of mixed inflammatory cells, resembling primary granulomas, and is separated from the surrounding normal parenchyma by a fibrous capsule (2). The underlying mechanisms of this phenomenon are still a subject of debate within the field of pathophysiology (25). However, the most widely accepted model proposes a central liquefaction of the necrotic centre, followed by erosion of the nearby airways and release of the liquefied contents resulting in the formation of an air-filled cavity (25). Whilst tuberculous bacilli do not multiply well in the cytoplasm of activated macrophages or in the solid caseous centres, both extracellular and intracellular bacilli can do so easily in an immunologically privileged location and be transmitted between individuals through aerosol spread (2, 14, 26). The advanced stage of post-primary disease in humans is likely the key aspect that animal models are unable to replicate in TB infections, creating a significant gap in our understanding of the disease (2). As well as the characteristics above, tissue hypoxia studies have revealed that areas of necrosis in pulmonary tissues from Mtb infected patients are developed in anaerobic conditions (17, 27–29). Hypoxia induces the secretion of matrix metalloproteinase-1 (MMP-1), which produce lung destruction mainly in areas of lung consolidation and around pulmonary cavities (29).

Lung samples from uncontrolled TB infections in humans are difficult to acquire so there is a lack of information regarding specific stages of granuloma development (17). For that reason, contrary to what happens in many TB animal models, there is not a clear classification of pulmonary granulomas from TB-infected humans. A first approach was performed by Canetti et al. (30), in 1955 and later by Ridley and Ridley (16) in 1987. Ridley and Ridley describe 6 groups of tuberculous granulomas: 1a, 1b, 2a, 2b, 3a and 3b, relating to the number of bacilli, the extension of the necrosis, and the cell composition. In samples from group 1a, neither necrosis nor AFB were present and mature epithelioid cells were the main cell type, whereas in samples from group 3b extensive necrosis, scanty macrophages and over 3 AFB per granuloma were observed (16). Fenhalls et al. (19) in 2002 classified the lesions from seven patients evaluated as necrotic and non-necrotic granulomas. Later, in 2016, Marakala et al. (18) described a different histopathological classification, including solid granulomas that lacked necrosis, caseous granulomas and cavitary granulomas. Further classification has been described by other authors, such as the following example for pulmonary granulomas: nascent, caseous, fibrocaseous and resolved granulomas (54).

## 3. Comparative pathology of animal models

### 3.1. Non-human primates

Old World monkeys, including the rhesus (*Macaca mulatta*) and cynomolgus (*Macaca fascicularis*) macaques, and New World monkeys (*Callithrix jacchus*, the common marmoset), are used as experimental models of human TB disease. They all develop the full range of Mtb disease observed in humans, including children and immunosuppressed patients, ranging from solid lesions to caseation, calcification, and cavitation (30, 56–59, 81). Each NHP model has benefits for certain types of studies; for instance, rhesus macaques have been shown to be more susceptible to Mtb infection compared with cynomolgus macaques who can show active as well as latent TB infection (58, 60). Depending on the strain of Mtb used, different rates of disease progression, as well as varying degrees of cavitation, are observed in the common marmoset (59). Two different outcomes of infection from Mtb have been seen in the cynomolgus macaque, depending on the challenge dose. It has been shown that when high doses of Mtb are administered, severe disease develops (acute TB) (55); conversely, a low-dose can result in asymptomatic infection which is similar to latent TB in humans (56, 61, 62).

Varying the route of infection does not result in different outcomes in terms of clinical parameters and behavioural changes between the macaque subspecies (63); however, lesion distribution does vary in the lung, with diffuse lesions more commonly associated with the aerosol route as compared to the intra-bronchial route (63, 64).

A range of pulmonary granulomas are observed in active TB in all NHP models. These range from early lesions consisting of small aggregates of immune cells, mainly macrophages with variable numbers of lymphocytes and neutrophils, and lacking clearly defined boundaries (65); the classical, organised, ‘solid’ structured granulomas which comprise abundant, epithelioid macrophages, neutrophils and some MNGCs in the absence of necrosis; granulomas that have a central core of necrosis but are not caseous and are surrounded by a rim of epithelioid macrophages and a peripheral rim of lymphocytes; and, large, well-defined, caseous granulomas (57, 62, 65). Cavitary lesions are rarely observed in the Old World NHP models during active disease (62); however, they have been noted in the common marmoset between 58 and 70-days post-challenge (dpc) when infected with the CDC1551 strain (59). TB pneumonia is also observed during active TB in all NHP models, and it is usually surrounding multicentric, often coalescing, large, caseous granulomas (59, 62). A summary of the main histopathological features in NHPs is represented in Table 1.

The majority of more advanced lesions mentioned above are also observed during latent TB infection (LTBI); however, granulomas without necrotic centres have not been noted. Large caseous granulomas are observed although the caseous core has often been replaced by mineralisation and/or collagenous material (62) defined as ‘fibro-calcified’ lesions by some groups (62). Also observed are lesions that are termed ‘sclerotic or fibrosing’ granulomas; lack visible mineralisation and comprise compacted, sclerotic material (62).

Depending on the strain of Mtb used and the species of NHPs, microscopic granulomas can be seen as early as 2–5 weeks post-challenge (wpc) (57, 65, 66). In rhesus macaques, early granulomas often lack clearly defined boundaries, but by 3 wpc, more ‘classical’ granulomas are seen (66). Central necrosis is also seen this timepoint when infected with H37Rv (65, 66). In cynomolgus macaques, however, the earliest manifestations of granulomas within the lung are present at 4–5 wpc, comprising multifocal to coalescing, caseous lesions (57). These granulomas are more like the ‘classical’ granuloma. A centre core of necrotic cells and amorphous material that is surrounded by a peripheral rim of epithelioid macrophages. In this zone, MNGCs may be observed where a number of macrophages have joined together to create a Langhan’s type giant cell (67, 68). In smaller, unorganised granulomas, MNGCs tend to be scattered throughout the lesion rather than in an outer rim (68). MNGC’s tend to be more abundant in the later, more advanced granulomas compared to the earlier manifestations (68). Beyond this there is rim of lymphocytes that consists of a mixture of B and T cells. It has been observed that as granulomas increase in size, B cell populations become more contained as ‘follicle-like’ structure in the lymphocyte rim, whereas in the smaller granulomas they tend to be scattered throughout this layer and the granuloma (15, 68–70). This is in contrast to findings from Fuller et al. (71) who state that B cells are only observed in follicle-like structures in caseous granulomas in cynomolgus macaques. Depending on the stage and extent of healing and containment of the granuloma a thin layer of fibrosis can develop around the external rim of lymphocytes (82). AFB are seen in most granulomas, but they are more abundant in larger granulomas with caseous necrosis (83).

**Figure 2:**
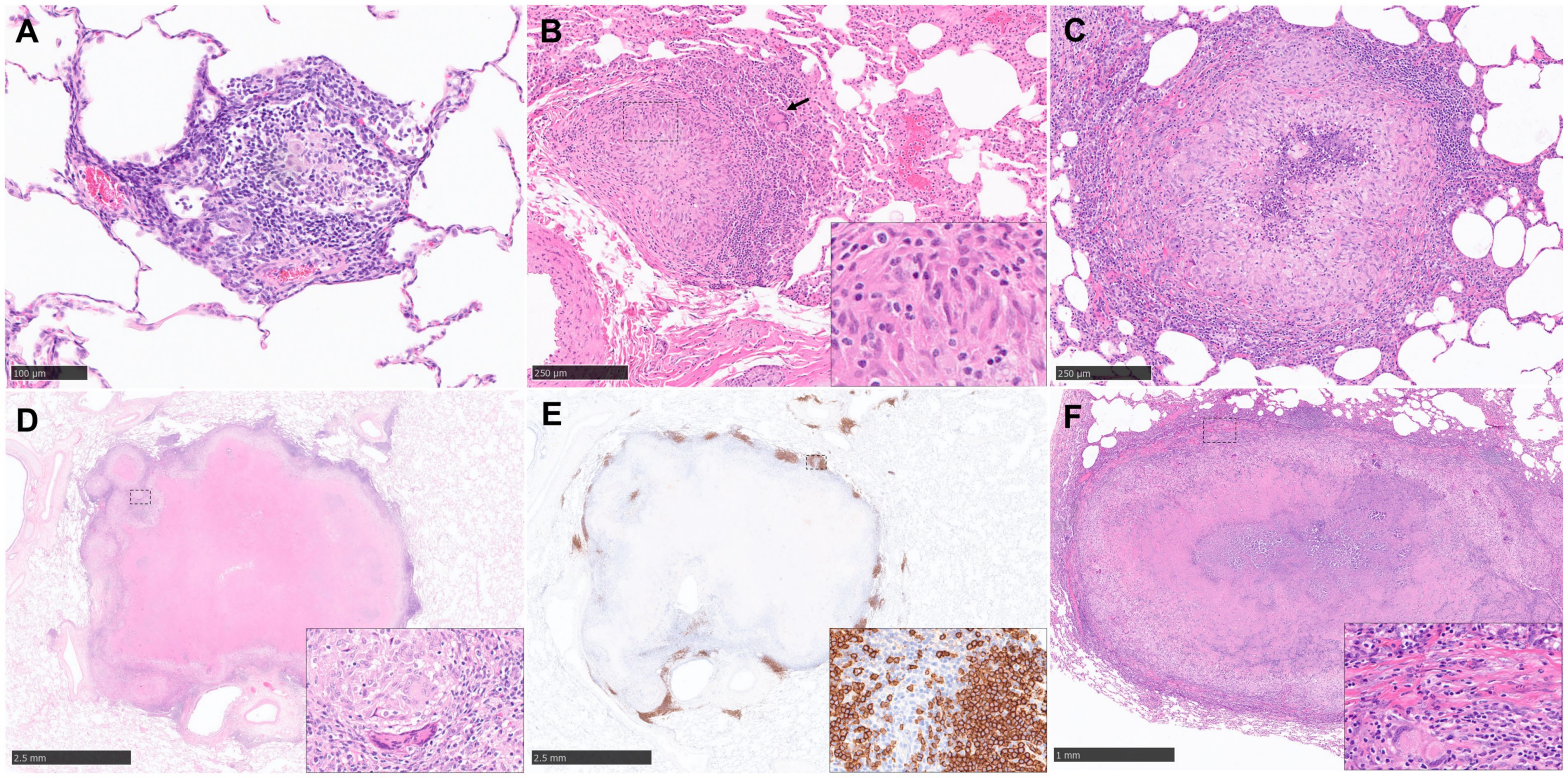
Representative histopathological images of the rhesus macaque (NHP) TB model in lung: (H&E) and CD20 staining (IHC). **(A)** Stage 1 granuloma characterized by the infiltration of the alveolar wall with macrophages and lymphocytes. **(B)** Stage 2 granuloma, well demarcated lesion composed by epithelioid macrophages (inset), macrophages and scattered MNGCs (arrow). **(C)** Stage 5 granuloma, larger lesion with a focal necrotic core composed of degenerated neutrophils and macrophages surrounded by viable cells. **(D, E)** Stage 6 granuloma, well demarcated lesion with central caseous necrosis surrounded by a layer of macrophages and an external rim of lymphocytes, with abundant B cells (CD20+) around the periphery forming “nests”. Inset shows some MNGCs **(D)** and CD20+ B cells **(E)**. **(F)** Late stage 6 granuloma, partially encapsulated by a fibrotic capsule (inset), characterized by the presence of a necrotic centre with dystrophic mineralization surrounded by viable immune cells. MNGCs are also present (inset). Scale bars = A, 100 µm; B and C, 250 µm; D and E, 2.5 mm; F, 1 mm.

The cellular composition of granulomas in NHPs used to distinguish between the type and severity of disease have been evaluated in various ways including semi-quantitative methods, for example, scoring systems (57, 65) as well as quantitative methods such as using image analysis to count the percentage of cells stained within a granuloma (31, 68). Scoring systems and lesion classifications have been used in NHP models in recent years and there are two that lead in the field; Lin et al. (57) whose system is based on 3 characteristics; the type of granulomas (caseous, solid, suppurative or mixed); the cellular composition (presence or absence of lymphocytic cuff, mineralisation, fibrosis, MNGCs and epithelioid macrophages) and the distribution pattern (focal, multifocal, coalescing and invasive). Lin et al. (57) have applied this to both active and latent TB experiments. The second is Rayner et al. (63, 65, 66) who published a more detailed description of the granulomas seen in active, early-stage TB infection which uses different stages of development to categorise the granulomas (stages 1–6). Briefly, stage 1 granulomas are small diffuse foci of cells including macrophages and lymphocytes and a few disperse neutrophils. They do not have clearly defined boundaries and they infiltrate alveolar walls and extend into the alveoli (Figure 2A). Stage 2 granulomas are unorganised but have a more defined boundary and they are larger than stage 1 but have the same mix of inflammatory cells (Figure 2B). Stage 3 granulomas are similar to stage 2, but they have a focal necrosis which is characterised by nuclear pyknosis and karyorrhexis with the loss of cellular architecture. Stage 4 lesions are circumscribed and primarily consist of macrophages mixed with neutrophils and other leucocytes and a few peripheral lymphocytes. Stage 5 granulomas are organised with a necrotic foci and degenerate neutrophils (Figure 2C). Finally, stage 6 is the classical, well demarcated granuloma that consists of central, caseous necrosis and has a variable rim of lymphocytes, mainly B cells, around the periphery (63, 65, 66) (Figures 2D–F). The use of this scoring system has been of great value evaluating vaccine candidates at the preclinical stage (32, 83).

### 3.2. Rodents

The mouse (*Mus musculus*) was one model utilised by Robert Koch to demonstrate that Mtb induced comparable lesions in mice to those observed in humans during natural infection (33, 34). The broad utilisation of mice in TB immunology research is attributed to the abundance of immunological tools and reagents, as well as the availability of inbred strains, which facilitate classical genetics and adoptive transfer experiments (13).

**Figure 3:**
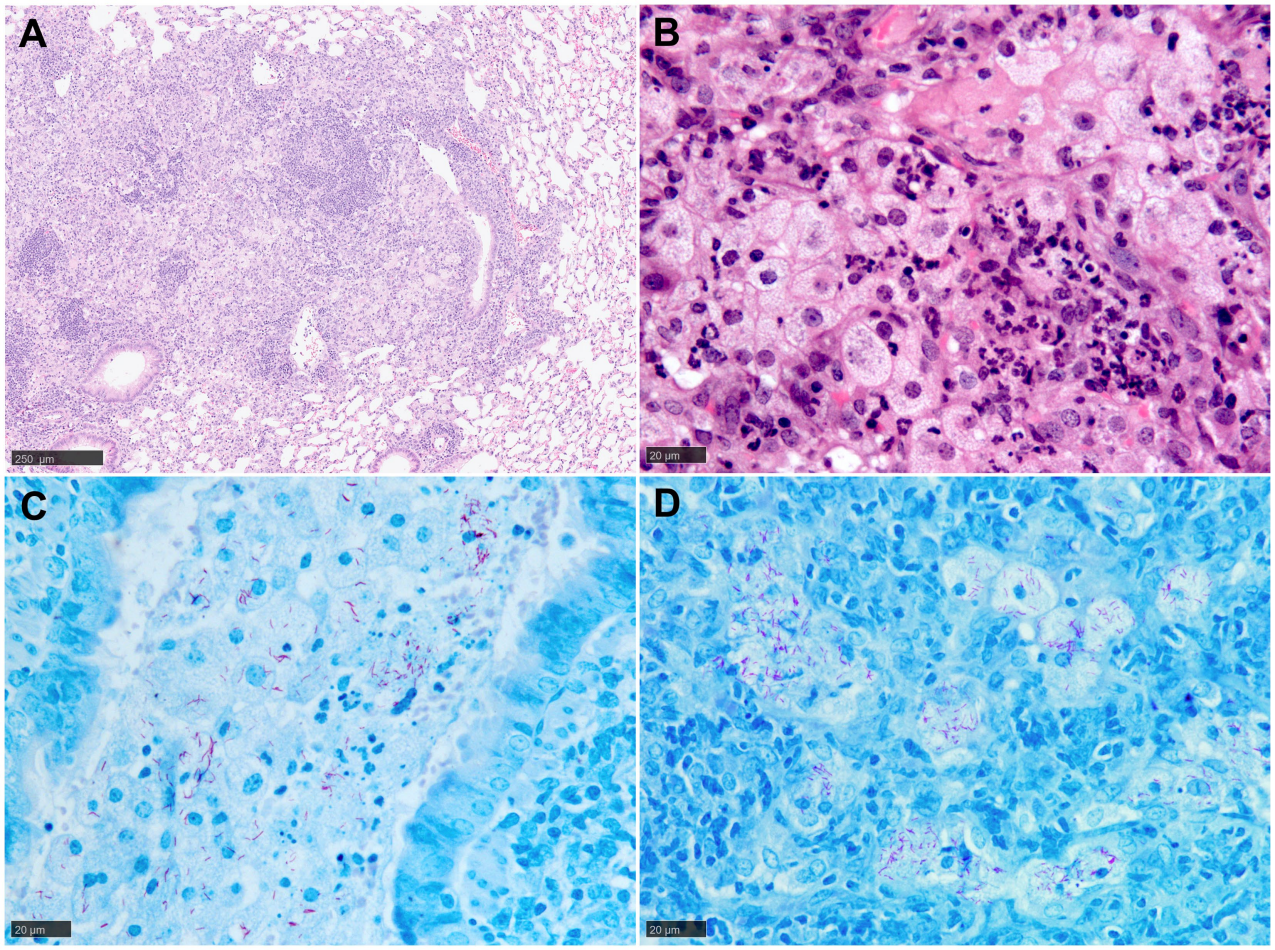
Representative histopathological images of the mouse (Balb/C) TB model in lung: (H&E) and Ziehl-Neelsen (ZN) staining. **(A)** Early-stage granuloma, non-demarcated and non-encapsulated, consisting mainly of macrophages, aggregates of lymphocytes and scattered polymorphonuclear cells. **(B)** Large foamy macrophages and polymorphonuclear cells in a late-stage granuloma. **(C)** Abundant AFB in the cytoplasm of foamy macrophages and cell debris within a bronchiolar lumen. **(D)** Large foamy macrophages with abundant intracellular AFB in a late-stage granuloma. Scale bars = A, 250 µm; B, C and D, 20 µm.

In general, mice tend to develop an acute rather than chronic infection with Mtb, and the granulomas formed in their lungs lack the structured and organised appearance observed in human granulomas (9) (Figure 3A). It should be noted that in most of the conventional strains of mouse models, granulomas do not develop the necrotic caseous centre, which is the primary characteristic of human TB (9, 13, 20, 35). Infected macrophages tend to show an enlarged cytoplasm with high lipidic content (foamy macrophages) that are interspersed with lymphocytes and neutrophils (Figure 3B). The presence of AFB is abundant within the macrophage cytoplasm and the necrotic cells (Figures 3C,D). Additionally, the lack of MNGCs in mice makes the tuberculous granulomas of mice histopathologically different from those observed in humans (17, 20, 36) (Figure 3). Table 1 summarises the main histopathological features in mice model observed during experimental pulmonary TB.

Numerous mouse models of infection have been developed, using mostly the intranasal infection route with the aim of mimicking pulmonary TB in humans as close to a natural infection route as possible (20, 24, 28, 36–41).

Rhoades et al. (20) infected C57BL/6 mice via the aerosol route with varying doses of three different Mtb strains and studied the development of a granuloma for a period of 1 year in the lung. Although the different conditions failed to induce a caseating necrosis, the mice developed a chronic form of pulmonary TB in all cases. Rhoades et al. (20) described five histopathological stages of granuloma development; category 1: small, isolated lesions diffusely distributed throughout infected lungs, composed of a few adjacent alveoli with thickened septae, consisting primarily of mononuclear phagocytes, alveolar macrophages and occasional lymphocytes; category 2: scattered, discrete foci of alveolitis filled with mononuclear phagocytes and a few epithelioid and foamy macrophages, with perivascular and peribronchiolar lymphocytes; category 3: moderate lesions characterised by sheets of epithelioid and foamy macrophages that fill the alveoli, with mild interstitial fibrosis and tight associations of lymphocytes. In this case, while evidence of irreversible cell degeneration and death was noticed, no central necrosis was detected. In category 4 enlarged, coalescing granulomatous lesions were detected, consisting mainly of the presence of macrophages, epithelioid macrophages, and large foamy macrophages. Small foci of necrosis and polymorphonuclear cells were associated with cleft of cholesterol and fibrosis of alveolar septae was more advanced. Finally, lesions in category five are defined as extensive chronic, interstitial fibrosing granulomas, characterised by thickened alveolar septae demarcated areas filled with dead/dying epithelioid and foamy macrophages (20). With high doses of Mtb, single AFB were detected by ZN stain in several alveolar macrophages and polymorphonuclear cells of category 2 granulomas after 20 dpc. Multiple AFB were more apparent after 60 dpc in epithelioid cells and foamy macrophages and no differences between categories 3 and 4 were found (20).

Cardona et al. (36) also used the same mouse model, although with different conditions, and they proposed a new histopathological approach in the development of pulmonary lesions: primary, secondary, and tertiary granulomas. Primary granulomas (category 3 in Rhoades et al. (20) classification) consisted of an initially poorly structured lesion with neutrophils, lymphocytes and infected macrophages surrounded by a thick mantle of lymphocytes, in which periphery foamy macrophages filled the alveolar spaces. By day 220 post-challenge, macrophages are the predominant cellular element at the core of granulomas. In secondary granulomas, small numbers of infected macrophages are surrounded by lymphocytes. On day 60 post-challenge, peripheral lymphocytes formed a thick mantle which is surrounded by foamy macrophages. Tertiary granulomas consist of several neighbouring granulomas coalescing and remain confined within the alveolar spaces and did not elicit any significant fibrous reaction. Tertiary granulomas could be considered as categories 2, 4 and 5 from Rhoades et al. (20) classification because they may represent primary and/or secondary granulomas linked with foamy macrophages (36). Although Cardona et al. (36) remarked on the absence of MNGCs and the scarcity of epithelioid cells, both authors agree that foamy macrophages are a fundamental cell in mouse tuberculous granulomas (20, 36). Indeed, the continued enlargement of granulomas is heavily reliant on the presence of this particular cell type, as its proliferation plays a major role in the merging of lesions and the subsequent formation of large-sized granulomas (36).

Tsai et al. (17) evaluated significant differences as well as common characteristics in lung granulomas from humans and C57BL/6 mice with TB. A common feature observed in both human and mouse granulomas was the presence of B lymphocyte aggregates (17). However, some differences were observed between the two species. While in mouse lungs B cell aggregates were surrounded by macrophages, in human tissues this was not observed. In the latter, a characteristic association of B cells and T cells was observed, with two different patterns of expression (17).

Other groups have used the Balb/C mouse model for the study of pulmonary tuberculosis (28, 39–41). This model showed two histopathological phases of the disease. The first one or acute phase was characterised by an infiltrate of inflammatory cells in the alveoli, blood vessel and bronchiole with the formation of granulomas. The second phase was chronic pneumonia characterised by focal necrosis (40). Using the same mouse strain, Irwin et al. (39) demonstrated similar lesions to category two described by Rhoades et al. (20) at 3 wpc which became multifocal to coalescing with the progression of the disease. No evidence of necrosis or fibrosis was described with this model (28, 39–42). Pan et al. (43) demonstrated that C3HeB/FeJ mice (commonly referred to as the Kramnik mouse model) displayed lung pathology with a central caseous necrosis and encapsulation, similar to what is observed in human tissues (43). Using the same model, it had been observed that these lesions were also hypoxic (pimidazole+ by IHC), another key feature of human TB lesion, as previously mentioned (27, 28). C3HeB/FeJ mice develop three different pulmonary lesions following aerosol infection; type I is the one that most resembles classical human TB granulomas. They become evident after 35–45 dpc and are characterised by acellular caseum in the centre of the lesion, karyorrhectic debris and a distinct band of darkly stained intact neutrophils. This band is surrounded by a rim of foamy macrophages and a fibrotic capsule (24, 28, 39, 41). In humans, the central core is composed primarily of macrophages and foamy macrophages, however, in this mouse model it appears to be composed mainly of neutrophils and, to a lesser extent, foamy macrophages (24). SYBR Gold acid-fast stain, revealed large number of intracellular bacilli in the foamy macrophages and intact neutrophils and extracellular bacilli within the necrotic caseum (24). The type II lesions in infected C3HeB/FeJ mice presented fulminant granulocytic pneumonia and type III were similar to lesions found in Balb/c mice following aerosol infection (24). Occasionally, this model demonstrated the development of central cavities, like the ones observed in human tuberculosis (28, 41). Recently, a ‘humanised’ mouse model generated by transplanted human foetal liver derived haematopoietic stem cells have been proposed (HIS-NSG mice) (42). TB granulomas in these HIS-NSG mice comprised solid non-necrotic granulomas, tuberculoid pneumonia, and caseous necrotic granulomas, having the ability to model human-like pulmonary granuloma initiation and formation (42).

In contrast to mice, the use of rats (*Rattus* spp.) for the study of human tuberculosis is less common. However, this species could also represent a good model due to the well documented immunology and the number of monoclonal antibodies which are commercially available (84). Studies before 2006 described lung granulomas as histologically similar to classic murine models, with the absence of central necrosis (21, 84, 85). In the Lewis rat strain, Sugawara et al. (85) described granulomas of various sizes from 3 wpc, observing foamy macrophages and MNGCs with the progression of the disease (84, 85). Sugawara et al. (84) described a model of TB in F344/N-rnu nude rat strain which developed granulomas with central necrosis and encapsulated by thick collagen fibres, mimicking those observed in humans. However, unlike humans, the central necrosis was not caseous (84).

The American cotton rat (*Sigmodon hispidus*) is a useful model in many human respiratory diseases such as respiratory syncytial virus (RSV) or influenza virus (86). In 2007, Elwood et al. also demonstrated its value as a model for pulmonary TB (21). Cotton rats developed a granulomatous inflammation, which was visible macroscopically, demonstrating multiple white nodules in the lung parenchyma. At 8 wpc, localised and discrete granulomas surrounded by healthy lung tissue and composed of epithelioid macrophages in the centre and lymphocytes at the periphery were observed. Caseous necrosis was frequently observed which became calcified with disease progression. Multiple AFB were noted within the cytoplasm of epithelioid cells and intracellular and extracellular in necrotic granulomas (21). Later, Singhal et al. proposed another model in Wistar rats endotracheally infected with different strains and doses of Mtb, which revealed some similarities with human TB (44). Histopathological analysis of lung from rats infected with high dose of W4 Mtb strain revealed granulomas with a central area of epithelioid macrophages and a cuff of lymphocytes, together with small peribronchial aggregates from 28 dpc onwards. At 60 dpc, well organised granulomas with many lymphocytes, foamy macrophages and occasional MNGCs were observed, although necrosis and fibrosis were seldom seen (44).

### 3.3. Guinea pigs

**Figure 4:**
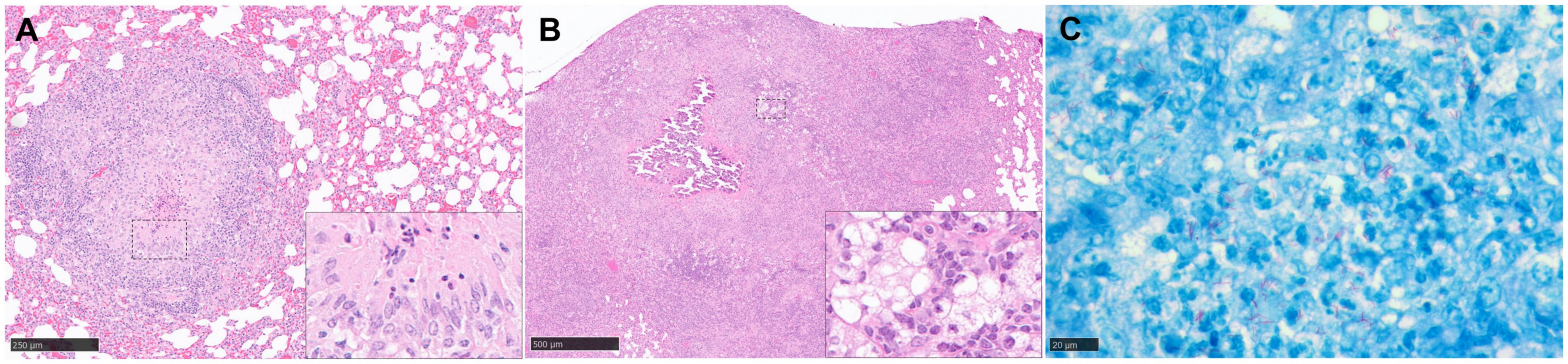
Representative histopathological images of the guinea pig TB model in lung: (H&E) and Ziehl-Neelsen (ZN) staining. **(A)** Stage III granuloma. Circumscribed and well demarcated granuloma, characterised by the presence of central necrosis surrounded by epithelioid macrophages and a peripheral rim of lymphocytes and plasma cells. Insets shows epithelioid macrophages and few necrotic heterophils. **(B)** Stage IV granuloma. Large non-encapsulated granuloma with central necrosis and dystrophic mineralisation, surrounded by epithelioid macrophages, foamy macrophages, and diffuse aggregates of lymphocytes. Inset shows foamy macrophages and lymphocyte aggregates. **(C)** Abundant intracellular and extracellular AFB in a stage II granuloma. Scale bars = A, 250 µm; B, 500 µm; C, 20 µm.

Guinea pigs have been used extensively in TB research; they share many features of disease with humans, making this species a popular choice as an animal model of TB model of human disease. The histopathological features of the TB lesions in the guinea pig, represented in Table 1 and Figure 4, have been well documented. Small lesions comprising mainly macrophages, with some neutrophils and lymphocytes, arise after a few days post aerosol challenge with Mtb. These lesions are often located close to large airways (45) and it has been suggested that these initial lesions are more likely to originate in the interstitium rather than alveolar spaces (46). This occurs through a process whereby bacilli escape from macrophages adhered to the alveolar epithelial surface and subsequently infiltrate into the surrounding, pulmonary tissue. Furthermore, neutrophils are thought to facilitate early lesion growth via degranulation and release of hydrolytic enzymes. At these early time points, the pulmonary lymphatics are also affected, with lymphangitis observed as early as 5–15 dpc and involving the connective tissue supporting delicate, peribronchial and perivascular, lymphatic vessels (47). These lymphangio-centric lesions continue to develop into granulomas in parallel with those located in the parenchyma. Macrophages, both epithelioid and foamy, and lymphocytes, continue to infiltrate lesions, resulting in larger, circumscribed ‘solid’ granulomas by days 15–20 post-challenge. As the granuloma enlarges, it compresses adjacent blood vessels, causing tissue hypoxia (48, 49). Although collagen deposition may form around the periphery of the granuloma as part of its progressive, structural development, fibrosis does not play as prominent a role as in other animal models such as NHPs or cattle with bTB; furthermore, pre-existing collagen in broncho-vascular connective tissue can be incorporated into the periphery of lesions that develop in these areas.

In addition, around days 15–20 post-challenge, signs of necrosis become evident; alongside this are increased numbers of granulocytes. Around this time point, the number of acid-fast bacilli observed in lesions also plateaus, coincident with the development of adaptive immunity (50).

This process rapidly progresses to prominent, central necrosis by around day 30 post-challenge (Figure 4A). It has been proposed that neutrophils are likely to be the key cells that initiate necrosis formation (46), as opposed to macrophage ‘softening’ and degradation. Dystrophic mineralisation, mainly calcification, of the necrotic core, a key feature in disease progression in this species, follows shortly afterwards (Figure 4B). Advanced staining techniques have identified numerous clumps of over 15 AFB within the necrotic core and its periphery, the latter of which is often observed as highly eosinophilic rim (51). Moreover, special stains have identified accumulation of extracellular ferric iron in areas of lesion necrosis, and iron accumulation in macrophages at the periphery of granulomas (52); this is commensurate with the requirement of Mtb for host iron to facilitate growth and promote increased virulence.

A prominent feature of pulmonary TB in guinea pigs is the formation of secondary lesions which may arise from a number of scenarios and reflect fundamental differences in host environments (53). Orme and Basaraba (46), propose that those located in a subpleural location may arise from the passive transportation of bacilli from primary lesions through pulmonary lymphatics towards the pleura where they lodge and develop lesions. Haematogenous spread can also result in secondary lung lesions; this has been demonstrated to occur within 2–3 wpc (53). As an adaptive immune response is likely to have established by the time secondary lesions are developing, their structure differs from their primary counterparts. In general, they are small, predominantly lymphocytic, lack the discrete granuloma morphology as well as neutrophils, necrosis and calcification; the number of intra-lesional bacilli are also reduced (53). This microscopic appearance is akin to primary lesions that form in the lungs of guinea pigs vaccinated with BCG prior to infection with Mtb (53); this results in partial protection from the adaptive immune response stimulated by the vaccine, and a reduction in the severity of the inflammatory response. Infection invariably leads to progressive disease, with the time taken to reach humane endpoints dependent on variables such as the mycobacterial strain and dose (87).

Many of the features described for guinea pigs are observed in TB infection in people. Both species develop large, pulmonary granulomas with central, caseating necrosis and surrounded by epithelioid macrophages, occasional MNGCs and a rim of lymphocytes and chronic, fibrous encapsulation (Table 1). Furthermore, prominent lymphadenopathy and lymphangitis are also shared features, as well as disease dissemination. Despite the paucity of other key morphological features noted in human disease, such as post-primary, cavitating lesions that facilitate rapid spread but that are observed uncommonly in the guinea pig, this species is considered the gold standard for the preclinical evaluation of both potential new vaccines and therapeutics (88).

Evaluation of differences in the type and severity of disease has traditionally seen the employment of semi-quantitative methods, such as subjective scoring systems (72, 73) which estimate extent of disease and numeration of particular features such as number of foci of necrosis or calcification. Quantitative analysis, determining percentage of lung affected by disease, such as consolidation, can also be estimated using proprietary software packages. Lesion classification systems have been devised to grade granuloma type in the lung of the guinea pig model (45). Our group has recently developed a new scoring system to differentiate morphological characteristics in a temporal pattern and which have been observed in both parenchyma and broncho-vascular, connective tissue (74). Briefly, stage I lesions incorporate small, poorly demarcated collections of inflammatory cells, primarily macrophages, with some lymphocytes and granulocytes. Larger, circumscribed and well demarcated, non-necrotic lesions comprising macrophages (mainly epithelioid), scattered lymphocytes and variable numbers of neutrophils, are considered stage II lesions; and these progress to stage III lesions when central necrosis is visible, often with a degree of caseation. The final stage, stage IV, contains granulomas with variable but often extensive, central, dystrophic calcification and caseating necrosis (Figure 4).

Reports on immunostaining of granulomas have enabled further characterisation of granulomas; Turner et al. (45) describe the lymphocytic population in the non-necrotic, solid granulomas, comprising of significant numbers of CD4+ cells with fewer CD8+ cells; interestingly, in contrast to mice, these cells were distributed randomly within the lesions without aggregating. By 30 dpc, numbers of these cell numbers decrease prominently and an increase in B cells and granulocytes is seen, with a concomitant worsening of pathology (75). More recently, our group have used a number of cell markers (CD3, CD4, Iba1, myeloperoxidase, Mac387) to describe presence and frequency of inflammatory and immune cells in granulomas up to 27 wpc. We have identified macrophages (Iba1+) as the predominant cell marker visible in all granuloma stages. By contrast, MAC387+ (calprotectin) staining in macrophages and granulocytes was noted in scattered granulomas, with an increase in staining correlating with lesion progression up to stage III and noted early in between weeks 3–4 post-challenge. Similar changes were reported for myeloperoxidase (MPO) in granulocytes, with particularly strong staining noted in stage IV granulomas between 8 and 27 wpc. Percentage of CD3+ staining was lowest in all granulomas at the early time point of 3 wpc, whilst maximum at 8 wpc, and similar expression of staining in all granuloma types noted at the later time points, up to 27 wpc. A progressive increase in B-cell marker quantification was observed from stage I to IV. Higher expression of this marker was detected at 8 wpc in comparison with weeks 4 and 24–27 wpc in stage I and II granulomas. As more markers become available for use in the guinea pig model, further characterisation is likely to occur.

### 3.4. Rabbits

Some consider the rabbit model good as a model of immunopathogenesis and rabbits can be experimentally infected with Mtb or *M. bovis* and they show similar characteristics as human TB disease, for example, caseous necrosis, liquefaction and cavitary disease (79, 80, 89, 90). Kaplan et al. also have developed a model of mycobacterial meningitis that closely resembles TB meningitis in people (90). The different strains of mycobacteria cause different manifestations of disease, for example, when infected with Mtb strain CDC1551, rabbits show similarities to latent TB infection when compared to humans as they can clear the bacilli and granulomas disappear (89). However, an infection with *M. bovis* creates a progressive TB disease that can lead to death (80, 91).

In the 1920’s, Lurie and their team bred rabbits that had different susceptibilities to Mtb strains, and these were used to look at the pathogenesis of TB in rabbits (92, 93). Unfortunately, these inbred rabbits no longer exist, and the outbred strains of rabbits tend to be resistant to Mtb. However, in 2004, Dorman et al. evaluated a new inbred strain of New Zealand White rabbits that appeared to be more susceptible to Mtb infection when compared to the outbred strains (79).

After experimental challenge, lesions can be seen as early as 2 wpc with increased cellularity within the alveolar spaces (89). The strain of Mtb used can affect the outcome of disease. CDC1551 strain has a different outcome when compared to HN878 strain and Erdman strain. Multiple granulomas are observed with strain HN878 at 4 wpc which consist of scattered lymphocytes, neutrophils, and macrophages. These lymphoid aggregates are often perivascular and peribronchial. MNGCs are frequently observed in macrophage abundant areas (89). However, by 4 wpc with strain CDC1551, small, scattered aggregates of cells are observed (89).Inbred rabbits have large granulomas with frequent caseous necrosis and obvious AFB by 5 wpc with Erdman strain, whereas in outbred rabbits the granulomas tend to be smaller with less caseous necrosis and few viable AFB (79). By 8 wpc with HN878 strain, granulomas are more abundant and central necrosis is present with abundant AFB (89), and in rabbits with CDC1551 infection the granulomas have increased in size, started to coalesce, and have become organised structures similar to the classical granulomas in humans; with a central core of necrosis, macrophage layer and an outer rim of lymphocytes (89, 94). At 12–16 wpc in HN878 infection, central necrosis increases, liquefaction can be seen in some granulomas, and cavities can also form. Some granulomas, however, have been observed to reduce in size an become mineralised. It has been noted that the mineralised granulomas have only a few AFB (94). At 12–16 wpc in CDC1551 infection, it has been observed that granulomas are organised and some have no AFB present. It is also not uncommon for granulomas to have been reabsorbed to a point where only increased cellularity in the parenchyma is seen (89).

Infection with Mtb Erdman strain produces coalescing granulomas with some lesions developing cavities at 16–18 wpc. It has been observed that some rabbits have ‘healed’ granulomas at this time point whilst others have a mixture of small and large granulomas (80). Rabbits challenged with *M. bovis* tend to have a chronic infection with TB pneumonia, fibrotic granulomas and cavitary disease (26, 91). These histological features are summarised in Table 1.

### 3.5. Ruminants

#### 3.5.1. Cattle

Bovine tuberculosis (bTB) is one of the most economically important disease in animal health worldwide. bTB is also a very important zoonosis and a major Public Health concern in low-income countries. For those reasons, bTB has been studied extensively and very valuable information about the pathogenesis of the disease and vaccination trails has been gathered over the past decades.

*M. bovis* is the main pathogen inducing bTB, and infected bovines (for example, *Bos taurus* and *Bos indicus*) show similar characteristics to Mtb infected humans (95). Other members of the MTBC can also infect bovines, e.g. *Mycobacterium caprae* showing very similar pathology when compared to *M. bovis* (96), or Mtb, showing a less pathogenic outcome in experimentally infected animals (6).

The main features of pulmonary granulomas in bTB are necrosis, mineralisation, a fibrous capsule, and presence of MNGCs (Table 1). AFB are present in higher numbers in more developed granulomas that show necrosis (5, 70, 95).

The earliest lesions described in lungs of experimentally infected cattle are at 15 dpc following an aerosol challenge with 1.12 × 10^4^ cfu (97). Granulomas at this timepoint are not organised. This early stage in granuloma development is characterised by an increase of perivascular cuffing of lymphocytes around lymph and blood vessels, an increase of macrophages as well as granulocytes within the congested interstitum (97). Palmer et al. (97) have described observing the alveoli filled with homogenous eosinophilic, fibrillar material that is consistent with oedema fluid and fibrin. Foamy macrophages are abundant, and a variable number of AFB are observed. No necrosis is observed at this stage. At day 30 post-challenge, as the infection progresses, these early lesions start to grow and accumulate more lymphocytes, macrophages, MNGCs and neutrophils which fill the alveolar spaces. Lymphocytes also form small, circle-like shapes. Some will have small areas of contained necrosis (97). After 90 days post-challenge, granulomas have become more organised structures.

Wangoo et al. (5) developed a scoring system for TB granulomas in cattle experimentally infected with *M. bovis* which many research groups have since used to evaluate their work. The scoring system characterises granulomas into 4 stages. Briefly, stage I (initial) granulomas consist of irregular clusters of epithelioid macrophages with few neutrophils and Langhan’s MNGCs with scattered lymphocytes, no necrosis observed at this stage. Stage II (solid) granulomas have either a complete or partial thin capsule. They primarily consist of epithelioid macrophages with minimal necrotic areas with infiltrates of lymphocytes, neutrophils and MNGCs as well as some haemorrhage. Stage III (minimal necrosis) granulomas are fully encapsulated, with central caseous necrosis and mineralisation. Surrounding the central necrosis are epithelioid macrophages and MNGCs. Towards the fibrous capsule, in the peripheral zone there are clusters of macrophages, lymphocytes, neutrophils and MNGCs. Finally, Stage IV (necrotic and mineralised) granulomas are large and irregular in shape, they are multicentric with prominent caseous necrosis. They have a thick capsule with an abundance of mineralisation which covers the majority of the lesion. MNGCs and epithelioid macrophages surround the necrotic core and there are dense clusters of lymphocytes towards the fibrotic capsule (5). Palmer et al. (97), looked at granulomas in cattle as early as day 15 and 30 post-challenge and proposed that the scoring system developed by Rayner et al. (65) for rhesus macaques was more suitable for these early timepoints than the commonly used Wangoo et al. (5) system.

There have been many studies that have looked at the cellular composition of bovine granulomas experimentally infected with *M. bovis*. Using techniques such as IHC and *in-situ* hybridisation (ISH) it has allowed us to characterise, visualise and locate different cell types within the granuloma (5, 7, 70, 97–99). CD68 is commonly used to identify macrophages, epithelial cells and MNGCs to help differentiate the different stages of granuloma development (70). CD68+ cells are most abundant in the early stages of lesion development (stage I and II) where they are scattered throughout the granuloma, whereas, by the more developed stages (III and IV) they are less abundant and are only located around the rim of the necrotic centre (70). IHC/ISH has allowed characterisation of MNGCs further by assessing the expression of cytokines including TNF-α, IFN-γ, TGF-β, IL-17A, and IL-10 (70, 100). From this characterisation it was observed that stage I early granulomas expressed more IL-17A and IL-10 when compared to late-stage granulomas (stage IV) (100). MNGCs have been observed in all studies of pulmonary granulomas infected with *M. bovis*, however, their role is still not fully understood. It has been suggested that they are used as a measure of lesion severity, with higher numbers associated with increased inflammation, more severe disease, and greater antigen persistence (101).

In the early stages of granulomas development (stages I/II) B cells tend to be scattered throughout the granuloma moving to follicle-like satellite nests around the outside of the fibrous capsule in the more advanced lesions (stages III/IV) (70, 102, 103). This is similar to what is seen with B cells during granuloma development in humans and NHPs (15, 68).

CD3+ T cells have been observed in a similar distribution to CD68+ cells as they are seen scattered throughout the lesion at stage I/II and then they are observed mainly in outer rim of the lesion at stage III/IV (70, 98). CD3+ T cells have further been identified to distinguish CD4+ T cells, CD8+ T cells and gamma/delta (γδ) T cells. This has shown that the predominant T cell subtype in all stage of granulomas is CD4+ T cells (98). Gamma/delta T cells appear to be predominantly in the early-stage granulomas with several studies showing that their presence decreases in the later stage of development (76, 98), however, some studies have shown that they are more abundant in the later stages of granuloma development (5, 70).

Neutrophils are thought to play an important role, especially in the early granuloma formation and are associated with the earliest signs of necrosis (104). Necrosis has been observed as early as 14–28 days post-challenge and studies have shown evidence of neutrophil infiltrates at day 14 (76, 104).

#### 3.5.2. Goats

Goats (*Capra hircus*) are considered valuable animal models and potential target species for studying TB due to their natural susceptibility to tuberculous mycobacteria such as *M. bovis, M. caprae* and Mtb (105, 106). Goat TB closely resembles bovine and human TB in terms of immune response and pathological characteristics. They exhibit comparable patterns of disease progression, with a predominant anatomical localisation within the respiratory tract and the formation of caseous granulomas (77, 107). In addition, goats demonstrate notable similarities in the anatomical structure of their respiratory tract, as well as a comparable size and body weight to that of humans (107). These shared features present a unique opportunity to bridge the gap between murine preclinical studies and subsequent human investigations (77, 105), thereby providing valuable insights into the intricate mechanisms of host-mycobacteria interaction (77).

In natural infections, as in cattle and humans, granulomas in goats are characterised by the presence of central caseous necrosis with varying degrees of mineralisation, surrounded by epithelioid macrophages, foamy macrophages, MNGCs, lymphocytes and a fibrotic capsule (78, 108, 109) (Table 1). Lymphocytes were mainly CD4+ T cells and B cells, located within the fibrotic capsule of the granuloma and the peripheric margin, respectively [reference (79)]. AFB are usually located inside the epithelioid macrophages and MNGCs [reference (79)]. Additionally, goats show a prominent tendency for the development of liquefactive necrosis and the formation of cavities within tuberculous granulomas, closely resembling the features observed in active TB cases in humans (78, 105, 108, 109). In the study performed by Sanchez et al. (78), the section of the cavitary lesions revealed the accumulation of intact and degenerate neutrophils and numerous intra- and extracellular AFB at the luminal surface [reference (79)]. The periphery was formed by granulation tissue with some macrophages and lymphocytes (mostly CD8+ T cells by IHC) and surrounded by a thick fibrotic capsule [reference (79)]. These cavitary lesions have been linked to high transmission rates, attributed to the higher abundance of AFB and a greater concentration of mycobacterial antigen detected within them (78, 109). These typical granulomatous lesions have also been reproduced in experimental conditions in goats, using low/middle doses (1,300–1,500 CFUs) of *M. bovis* or *M. caprae* through various inoculation routes, including intratracheal (105), transthoracic (110), and endobronchial (107, 108).

The gross pathology and histopathological scoring system developed by Vordermeier et al. (111) in 2002 and Wangoo et al. (5) in 2005 have also been adapted for use in goats (108, 112–115). The macroscopic evaluation categorises the pulmonary lung lesions in four different groups according to the percentage of the lobe affected, being 0 no evident TB-compatible lesions and 4 > 75% of the percentage affected (112, 113). Another gross approximation involves the meticulous sectioning of lung and lung-associated lymph nodes into thin sections measuring 0.5 to 4 mm in thickness. Through thorough observation, this approach allows for a semi-quantitative assessment of lesion size and distribution (108, 114). Histologically, the four granuloma developmental stages are the ones described in bovine: stage I (initial), stage II (solid), stage III (minimal necrosis) and stage IV (necrosis and mineralisation). Moreover, the detection of mycobacteria and the identification and quantification of AFB are conducted using IHC and ZN staining, as in the other animal species and in human beings (78, 96, 109, 116, 117). This standardised approach facilitates the quantification and characterisation of lesions, enabling consistent comparisons across different individuals and research studies. IHC has also been widely used to characterise the course of pulmonary TB infection in goats detecting the mycobacteria or host immune markers, including macrophages, neutrophils, T and B cells (78, 117, 118) within the lesions, providing insights into the host-pathogen interactions and local immune response during TB infection.

#### 3.5.3. Other ruminants

The infection by *M. bovis* has been extensively described in domestic and wild ruminants of importance for animal or public health, mostly linked to bTB (95). The in-depth study of the pathology and pathogenesis of *M. bovis* infection in these species has been of great value to understand the dynamics of infection in natural and experimental conditions. We have described the goat model as a good model for some pathological features of human TB such as the liquefactive necrosis and the formation of cavernous lesions (105). In contrast, sheep (*Ovis aries*), considered a rare host for MTBC infection, show lesions similar to those observed in cattle infected with *M. bovis*. Sheep have been used experimentally to evaluate vaccine efficacy against the infection (119).

In contrast, cervids with natural MTBC infection show more caseous granulomas with a very soft centre that can resemble abscesses macroscopically; these caseous lesions show histologically a central necrotic core, an outer layer of lymphocytes, activated macrophages, abundant MNGCs and AFB and, in many occasions, poor encapsulation (120).

White-tailed deer (*Odocoileus virginianus*) can be naturally infected with *M. bovis* and this has been replicated in experimentally challenged white-tailed deer. Whilst the lymph nodes are the most common organ to observe tuberculous granulomas in this species due to the route of natural transmission, the lungs of a small percentage of deer also show tuberculous granulomas (121). When experimentally challenged via the intratonsilar route with *M. bovis* strain 1,315, lungs lesions are first observed 42 dpc. According to a study by Palmer et al. (121), the left caudal, right middle and caudal lobes were observed to be the main lung lobes affected. Granulomas were often small, solitary and approximately 3 – 10 mm in size. Granulomas by 56 and 89 dpc showed some mineralisation of the necrotic core as well as some evidence of mild peripheral fibrosis. Liquefaction and abscess-like centres of lesions have been commonly observed in lymph nodes however, this has not been observed in lung lesions (121). Palmer et al. (121), have observed that lung lesions follow a similar development as lymph node lesions in this species. MNGCs can be observed from 42 dpc in the lung with low numbers of AFB present in lesions.

### 3.6. Zebrafish

Over the past few decades, the zebrafish (*Danio rerio*) has emerged as a widely utilised alternative vertebrate animal model in the study of mycobacterial disease (122). This species can be infected with *Mycobacterium marinum* (*M. marinum*), a genetically close relative of MTBC and a natural pathogen affecting poikilothermic species, in which it induces a systemic TB-like disease (106). Fish mycobacteriosis, resembling human TB, can manifest as either an acute infection or a chronic progressive disease characterised by the containment of mycobacteria in well-organised granulomas structurally similar to those caused by Mtb in humans (123–125). Remarkably, despite lacking lungs, zebrafish exhibit high genomic homology with humans, and possess innate and adaptive immunity systems that are comparable to mammals (122, 126, 127). Furthermore, this species displays disease phases observed in humans, including latency and reactivation (125). These shared features make zebrafish an exceptional model for understanding the disease progression and host responses involved (122, 126, 127).

In the natural environment, *M. marinum* spreads through water and primarily enters the host through the oral route, whereas in experimental conditions, zebrafish are commonly infected through intraperitoneal or intramuscular inoculation (124, 128, 129). The initial mycobacterial dose plays a critical role in determining the outcome of infection (125). Parenteral high-doses (>500 CFUs) lead to a more progressive and active disease with higher bacterial burden and mortality rates, while lower doses (~5–90 CFUs) can result in a chronic process with latent disease characterised by a stable numbers of granulomas (124, 128, 129). Following a successful *M. marinum* challenge, both larval and adult zebrafish models develop granulomas consistent with those reported in human TB, which can be found in various organs such as pancreas, gonads, kidney, liver, and occasionally the brain (106, 129). In embryos and larvae, early granuloma formation initiated by the innate immune response, can be visualised using *in vivo* real-time imaging within a few days post-challenge due to their transparency. These early lesions primarily consist of aggregated macrophages, few recruited neutrophils, epithelioid cells and MNGCs surrounding mycobacteria (123, 125, 130, 131). In adult zebrafish, initial TB-like granulomas characterised by cellular and bacterial aggregation have been reported at 2 wpc using both high and low infection doses. In advanced stages of the disease (16–20 wpc), mature granulomas are observed, featuring a caseous necrotic core surrounded by infected macrophages, epithelioid cells, neutrophils, infiltrating T and B lymphocytes, and a fibrous capsule (124, 129, 132–135). However, unlike mammalian tuberculous granulomas, zebrafish lesions contain fewer lymphocytes, and the presence of mineralisation has not been reported (124, 129, 131). Table 1 summarises the main histopathological features in zebrafish model observed during experimental pulmonary TB.

Various evaluation methods have been employed to study the TB-like lesions in zebrafish, enabling a comprehensive analysis of disease progression and the involvement of innate and adaptive immune responses. The transparency exhibited during the embryo and larval stages, offers a distinctive feature for investigating the initial stages of mycobacterial pathogenesis *in vivo* (131). Several authors have employed advanced *in vivo* real-time imaging techniques, including the utilisation of diverse immunofluorescence techniques to mark leukocytes and macrophages *in situ* (130, 136, 137). These techniques have demonstrated the ability of embryonic macrophages to phagocytose *M. marinum* within 1 h post-challenge, followed by the development of early granulomatous lesions 3–4 dpc (136).

Histopathological characterisation of the lesions has also been described using conventional histopathology and scoring systems. Risalde et al. (135) and López et al. (138) classified the granulomas into four histopathological stages based on their extent and cellular composition. Type I involved an infiltrate of epithelioid macrophages surrounding scattered mycobacteria, without necrotic areas or fibrous capsules. Type II represented partially encapsulated granulomas with a cluster of epithelioid macrophages and initial signs of necrosis. Type III consisted of encapsulated and well-organised granulomas containing regions of partial and complete necrosis, along with the presence of AFB. Type IV depicted encapsulated granuloma with an extensive necrotic core with a high mycobacteria concentration. Additionally, ZN staining revealed the presence of AFBs in both low and high infection dose experiments, primarily associated to necrotic areas (129, 132, 135, 138). In this context, Risalde et al. (135) and López et al. (138) developed a semi-quantitative assessment method to evaluate the presence of AFBs within the granulomas, categorised as: absent (0), very scarce (1–9), scarce (10–29), moderate (30–36, 54–71, 81–83), intense (36–53, 72–75, 84–100), very intense (>100).

Although the zebrafish has its limitations as a TB animal model, its distinctive characteristics have made significant contributions to our understanding of TB and mycobacterial disease in general. By providing valuable insights into host-pathogen interactions, immune responses, and the complex dynamics of TB granuloma formation, the zebrafish model serves as a crucial bridge between *in vitro* cell cultures and mammalian models, facilitating the translation of research findings to human clinical trials.

### 3.7. Other animal models

Many other animal species can be infected by MTBC bacteria. Natural TB in wildlife species such as badgers and wild boar (*Sus scrofa*) have been extensively studied due to its epidemiological importance in bTB, representing major reservoirs of infection for domestic livestock (139).

The histopathological features of granulomas in wild boar and domestic swine are similar to those observed in cattle and the differentiation of granulomas by developmental stages (stages I-IV) has been used in this species (140). Interestingly, wild boar can also show small lesions with heavy mineralisation and thick encapsulation, with very few immune cells and AFBs (95). The scoring system used for pulmonary lesion characterisation has been very valuable in this species to study the effect of different therapies against MTBC infection (141). Minipigs (*Sus domesticus*) have also been proposed as an excellent experimental model for the study of human TB, mimicking some histopathological characteristics observed in humans, such as granuloma encapsulation, and other parameters related with the immune response (142).

The Eurasian badger (*Meles meles*) is the main reservoir for bTB in the British Isles (143), and have been targeted for experimental vaccination to reduce the prevalence of bTB in these territories. The pulmonary lesions in naturally or experimentally infected badgers are typically solid, composed mainly of epithelioid macrophages with fewer lymphocytes, no apparent MNGCs and limited areas of necrosis and mineralisation (144). BCG has been licensed as a vaccine for TB in badgers after extensive experimental work, having used a version of the four developmental stages of the granulomas described by Wangoo et al. (5) in 2005 as a base for a histopathological scoring system principles (145, 146).

Following the 3Rs recommendations (replacement, reduction, and refinement), other less-conventional animal models have been used to study MTBC infection. For example, wax moth (*Galleria mellonella*) larvae, a well characterised model of infection for enterobacteria (147) has been recently used to study Mtb infection (148). It has been shown that phagocytic haemocytes can show internalisation of AFBs and cell aggregation towards a rudimentary ‘granuloma-like’ structure. Even though the model does not show many features of the typical pathology after mycobacterial infection, it can serve as a valuable tool to study the innate responses and identifying MTBC virulence genes (149). Moreover, novel *in vitro* models like the lung-on-chips are being developed to study the MTBC pathogenesis and will serve in the future to screen potential therapies against the disease reducing the number of animals used (150).

*M. orygis* has also been isolated in both humans and animals in S Asia and India. There are ongoing studies to evaluate whether this MTBC subspecies is a proxy for zoonotic TB. Currently, there is no consensus as to whether *M. orygis* should be identified as zoonotic TB along with *M. bovis* which is presently the only zoonotic MTBC subspecies defined by the WHO (151).

## 4. Conclusion and future considerations

TB continues to be a major Public Health concern in the post-COVID19 era. The emergence of multidrug resistant strains and the high prevalence in immunosuppressed individuals, mainly due to HIV infection, makes TB one of the top ranked infectious diseases causing death in the human population. The only licensed vaccine against TB is the BCG, with a variable degree of protection. Many new vaccines are in the preclinical and clinical pipeline with the hope to have novel tools to control this disease. Preclinical animal models are crucial to evaluate the safety and efficacy of these new vaccine candidates, as well as new therapeutics.

The in-depth knowledge of the pathology and pathogenesis of TB infection in these animal models has been of great value to advance in the fight against TB. However, these data should be considered carefully in the context of this disease and more studies are necessary to establish the best and most appropriate model for the study of TB, and to carry out a standard characterisation and score of pulmonary lesions.

With the development of new molecular tools used in pathology like multiplex staining linked to quantitative analysis or spatial transcriptomics, new valuable knowledge will be gathered from these animal models of TB and the new generation of alternative models to reduce and replace animals following the 3Rs.

## Data availability statement

The original contributions presented in the study are included in the article. Further inquiries can be directed to the corresponding author.

## Ethics statement

All animal studies reviewed in this article were reviewed and approved by the correspondent Animal Welfare and Ethical Review body.

## Author contributions

LH: Conceptualization, Writing – original draft, Writing – review & editing. IR-T: Writing – original draft, Writing – review & editing. IA-R: Writing – original draft. ER: Writing – original draft. FS: Conceptualization, Writing – original draft, Writing – review & editing.

## References

[1] WHO. Tuberculosis Fact Sheet (2023). Available from: https://www.who.int/news-room/fact-sheets/detail/tuberculosis.

[2] Basaraba RJ Hunter RL. Pathology of tuberculosis: how the pathology of human tuberculosis informs and directs animal models. Microbiol Spectr. (2017) 5. doi: 10.1128/microbiolspec.TBTB2-0029-2016 PMC11687511 28597826

[3] Hunter RL Jagannath C Actor JK. Pathology of Postprimary tuberculosis in humans and mice: contradiction of long-held beliefs. Tuberculosis (Edinb). (2007) 87:267–78. doi: 10.1016/j.tube.2006.11.003 17369095

[4] Ravimohan S Kornfeld H Weissman D Bisson GP. Tuberculosis and lung damage: from epidemiology to pathophysiology. Eur Respir Rev. (2018) 27:2017. doi: 10.1183/16000617.0077-2017 PMC6019552 29491034

[5] Wangoo A Johnson L Gough J Ackbar R Inglut S Hicks D. Advanced granulomatous lesions in *Mycobacterium Bovis*-infected cattle are associated with increased expression of type I procollagen, Gammadelta (Wc1+) T cells and cd 68+ cells. J Comp Pathol. (2005) 133:223–34. doi: 10.1016/j.jcpa.2005.05.001 16154140

[6] Villarreal-Ramos B Berg S Whelan A Holbert S Carreras F Salguero FJ. Experimental infection of cattle with *Mycobacterium Tuberculosis* isolates shows the attenuation of the human tubercle Bacillus for cattle. Sci Rep. (2018) 8:894. doi: 10.1038/s41598-017-18575-5 29343690 PMC5772528

[7] Salguero FJ Gibson S Garcia-Jimenez W Gough J Strickland TS Vordermeier HM. Differential cell composition and cytokine expression within lymph node granulomas from Bcg-vaccinated and non-vaccinated cattle experimentally infected with *Mycobacterium Bovis*. Transbound Emerg Dis. (2017) 64:1734–49. doi: 10.1111/tbed.12561 27615603

[8] Palmer MV Kanipe C Boggiatto PM. The bovine Tuberculoid granuloma. Pathogens. (2022) 11:1. doi: 10.3390/pathogens11010061 PMC8780557 35056009

[9] Silva Miranda M Breiman A Allain S Deknuydt F Altare F. The tuberculous granuloma: an unsuccessful host Defence mechanism providing a safety shelter for the Bacteria? Clin Dev Immunol. (2012) 2012:139127. doi: 10.1155/2012/139127 22811737 PMC3395138

[10] Donald PR Diacon AH Thee S. Anton Ghon and his colleagues and their studies of the primary focus and complex of tuberculosis infection and their relevance for the twenty-first century. Respiration. (2021) 100:557–67. doi: 10.1159/000509522 33321506

[11] Ulrichs T Kaufmann SH. New insights into the function of granulomas in human tuberculosis. J Pathol. (2006) 208:261–9. doi: 10.1002/path.1906 16362982

[12] Cronan MR. In the thick of it: formation of the tuberculous granuloma and its effects on host and therapeutic responses. Front Immunol. (2022) 13:820134. doi: 10.3389/fimmu.2022.820134 35320930 PMC8934850

[13] Ramakrishnan L. Revisiting the role of the granuloma in tuberculosis. Nat Rev Immunol. (2012) 12:352–66. doi: 10.1038/nri3211 22517424

[14] ScribaTJCoussensAKFletcherHAJacobsWRJrMcShaneHMizrahiV. Human immunology of tuberculosis. Microbiology. Spectrum. (2017) 5:1. doi: 10.1128/microbiolspec.TBTB2-0016-201628155806

[15] Ulrichs T Kosmiadi GA Trusov V Jörg S Pradl L Titukhina M. Human tuberculous granulomas induce peripheral lymphoid follicle-like structures to orchestrate local host Defence in the lung. J Pathol. (2004) 204:217–28. doi: 10.1002/path.1628 15376257

[16] Ridley DS Ridley MJ. Rationale for the histological Spectrum of tuberculosis. A basis for classification. Pathology. (1987) 19:186–92. doi: 10.3109/00313028709077132 3453999

[17] Tsai MC Chakravarty S Zhu G Xu J Tanaka K Koch C. Characterization of the tuberculous granuloma in murine and human lungs: cellular composition and relative tissue oxygen tension. Cell Microbiol. (2006) 8:218–32. doi: 10.1111/j.1462-5822.2005.00612.x 16441433

[18] Marakalala MJ Raju RM Sharma K Zhang YJ Eugenin EA Prideaux B. Inflammatory signaling in human tuberculosis granulomas is spatially organized. Nat Med. (2016) 22:531–8. doi: 10.1038/nm.4073 27043495 PMC4860068

[19] Fenhalls G Stevens L Moses L Bezuidenhout J Betts JC Helden Pv P. In situ detection of *Mycobacterium Tuberculosis* transcripts in human lung granulomas reveals differential gene expression in necrotic lesions. Infect Immun. (2002) 70:6330–8. doi: 10.1128/IAI.70.11.6330-6338.2002 12379712 PMC130373

[20] Rhoades ER Frank AA Orme IM. Progression of chronic pulmonary tuberculosis in mice Aerogenically infected with virulent *Mycobacterium Tuberculosis*. Tuber Lung Dis. (1997) 78:57–66. doi: 10.1016/s0962-8479(97)90016-2 9666963

[21] Elwood RL Wilson S Blanco JC Yim K Pletneva L Nikonenko B. The American cotton rat: a novel model for pulmonary tuberculosis. Tuberculosis (Edinb). (2007) 87:145–54. doi: 10.1016/j.tube.2006.07.001 16973421

[22] Wiley EL Mulhollan TJ Beck B Tyndall JA Freeman RG. Polyclonal antibodies raised against Bacillus Calmette-Guerin, Mycobacterium Duvalii, and *Mycobacterium Paratuberculosis* used to detect mycobacteria in tissue with the use of Immunohistochemical techniques. Am J Clin Pathol. (1990) 94:307–12. doi: 10.1093/ajcp/94.3.307 1697733

[23] Ulrichs T Lefmann M Reich M Morawietz L Roth A Brinkmann V. Modified Immunohistological staining allows detection of Ziehl-Neelsen-negative *Mycobacterium Tuberculosis* organisms and their precise localization in human tissue. J Pathol. (2005) 205:633–40. doi: 10.1002/path.1728 15776475

[24] Irwin SM Driver E Lyon E Schrupp C Ryan G Gonzalez-Juarrero M. Presence of Multiple lesion types with vastly different microenvironments in C3heb/Fej mice following aerosol infection with *Mycobacterium Tuberculosis*. Dis Model Mech. (2015) 8:591–602. doi: 10.1242/dmm.019570 26035867 PMC4457037

[25] Ihms EA Urbanowski ME Bishai WR. Diverse cavity types and evidence that mechanical action on the necrotic granuloma drives tuberculous cavitation. Am J Pathol. (2018) 188:1666–75. doi: 10.1016/j.ajpath.2018.04.006 29753789 PMC6109696

[26] Converse PJ Dannenberg AM Estep JE Sugisaki K Abe Y Schofield BH. Cavitary tuberculosis produced in rabbits by aerosolized virulent tubercle Bacilli. Infect Immun. (1996) 64:4776–87. doi: 10.1128/iai.64.11.4776-4787.1996 8890239 PMC174445

[27] Harper J Skerry C Davis SL Tasneen R Weir M Kramnik I. Mouse model of necrotic tuberculosis granulomas develops hypoxic lesions. J Infect Dis. (2012) 205:595–602. doi: 10.1093/infdis/jir786 22198962 PMC3266133

[28] Driver ER Ryan GJ Hoff DR Irwin SM Basaraba RJ Kramnik I. Evaluation of a mouse model of necrotic granuloma formation using C3heb/Fej mice for testing of drugs against *Mycobacterium Tuberculosis*. Antimicrob Agents Chemother. (2012) 56:3181–95. doi: 10.1128/AAC.00217-12 22470120 PMC3370740

[29] Belton M Brilha S Manavaki R Mauri F Nijran K Hong YT. Hypoxia and tissue destruction in pulmonary Tb. Thorax. (2016) 71:1145–53. doi: 10.1136/thoraxjnl-2015-207402 27245780 PMC5136721

[30] Canetti G. The tubercle Bacillus in the pulmonary lesion of man: Histobacteriology and its bearing on the therapy of pulmonary tuberculosis. New York: Springer Publishing Company (1955).

[31] Wong EA Joslyn L Grant NL Klein E Lin PL Kirschner DE. Low levels of T cell exhaustion in tuberculous lung granulomas. Infect Immun. (2018) 86. doi: 10.1128/iai.00426-18 PMC6105875 29891540

[32] White AD Sibley L Gullick J Sarfas C Clark S Fagrouch Z. Tb and Siv coinfection; a model for evaluating vaccine strategies against Tb reactivation in Asian origin Cynomolgus macaques: a pilot study using Bcg vaccination. Vaccines (Basel). (2021) 9. doi: 10.3390/vaccines9090945 PMC8473354 34579182

[33] Orme IM Collins FM. Mouse model of tuberculosis. in Tuberculosis. Ed. Bloom BR (1994):111–34.

[34] Gupta UD Katoch VM. Animal models of tuberculosis. Tuberculosis (Edinb). (2005) 85:277–93. doi: 10.1016/j.tube.2005.08.008 16249122

[35] Cosma CL Sherman DR Ramakrishnan L. The secret lives of the pathogenic mycobacteria. Annu Rev Microbiol. (2003) 57:641–76. doi: 10.1146/annurev.micro.57.030502.091033 14527294

[36] Cardona PJ Llatjos R Gordillo S Diaz J Ojanguren I Ariza A. Evolution of granulomas in lungs of mice infected Aerogenically with *Mycobacterium Tuberculosis*. Scand J Immunol. (2000) 52:156–63. doi: 10.1046/j.1365-3083.2000.00763.x 10931383

[37] Huynh KK Joshi SA Brown EJ. A delicate dance: host response to mycobacteria. Curr Opin Immunol. (2011) 23:464–72. doi: 10.1016/j.coi.2011.06.002 21726990

[38] Gonzalez-Juarrero M Turner OC Turner J Marietta P Brooks JV Orme IM. Temporal and spatial arrangement of lymphocytes within lung granulomas induced by aerosol infection with *Mycobacterium Tuberculosis*. Infect Immun. (2001) 69:1722–8. doi: 10.1128/iai.69.3.1722-1728.2001 11179349 PMC98078

[39] Irwin SM Gruppo V Brooks E Gilliland J Scherman M Reichlen MJ. Limited activity of Clofazimine as a single drug in a mouse model of tuberculosis exhibiting Caseous necrotic granulomas. Antimicrob Agents Chemother. (2014) 58:4026–34. doi: 10.1128/AAC.02565-14 24798275 PMC4068578

[40] Hernández-Pando R Orozcoe H Sampieri A Pavón L Velasquillo C Larriva-Sahd J. Correlation between the kinetics of Th1, Th2 cells and pathology in a murine model of experimental pulmonary tuberculosis. Immunology. (1996) 89:26–33. 8911136 PMC1456655

[41] Lanoix JP Lenaerts AJ Nuermberger EL. Heterogeneous disease progression and treatment response in a C3heb/Fej mouse model of tuberculosis. Dis Model Mech. (2015) 8:603–10. doi: 10.1242/dmm.019513 26035868 PMC4457036

[42] Arrey F Lowe D Kuhlmann S Kaiser P Moura-Alves P Krishnamoorthy G. Humanized mouse model mimicking pathology of human tuberculosis for in vivo evaluation of drug regimens. Front Immunol. (2019) 10:89. doi: 10.3389/fimmu.2019.00089 30766535 PMC6365439

[43] Pan H Yan BS Rojas M Shebzukhov YV Zhou H Kobzik L. Ipr1 gene mediates innate immunity to tuberculosis. Nature. (2005) 434:767–72. doi: 10.1038/nature03419 15815631 PMC1388092

[44] Singhal A Aliouat el M Herve M Mathys V Kiass M Creusy C. Experimental tuberculosis in the Wistar rat: a model for protective immunity and control of infection. PloS One. (2011) 6:e18632. doi: 10.1371/journal.pone.0018632 21533270 PMC3075263

[45] Turner OC Basaraba RJ Orme IM. Immunopathogenesis of pulmonary granulomas in the Guinea pig after infection with *Mycobacterium Tuberculosis*. Infect Immun. (2003) 71:864–71. doi: 10.1128/IAI.71.2.864-871.2003 12540568 PMC145351

[46] Orme IM Basaraba RJ. The formation of the granuloma in tuberculosis infection. Semin Immunol. (2014) 26:601–9. doi: 10.1016/j.smim.2014.09.009 25453231

[47] Basaraba RJ Smith EE Shanley CA Orme IM. Pulmonary lymphatics are primary sites of *Mycobacterium Tuberculosis* infection in Guinea pigs infected by aerosol. Infect Immun. (2006) 74:5397–401. doi: 10.1128/IAI.00332-06 16926435 PMC1594862

[48] Lenaerts AJ Hoff D Aly S Ehlers S Andries K Cantarero L. Location of persisting mycobacteria in a Guinea pig model of tuberculosis revealed by R207910. Antimicrob Agents Chemother. (2007) 51:3338–45. doi: 10.1128/aac.00276-07 17517834 PMC2043239

[49] Via LE Lin PL Ray SM Carrillo J Allen SS Eum SY. Tuberculous granulomas are hypoxic in Guinea pigs, rabbits, and nonhuman Primates. Infect Immun. (2008) 76:2333–40. doi: 10.1128/IAI.01515-07 18347040 PMC2423064

[50] Creissen E Izzo L Dawson C Izzo AA. Guinea pig model of *Mycobacterium Tuberculosis* infection. Current Protocols. (2021) 1:e312. doi: 10.1002/cpz1.312 34941021

[51] Ryan GJ Hoff DR Driver ER Voskuil MI Gonzalez-Juarrero M Basaraba RJ. Tuberculosis phenotypes in mouse and Guinea pig lung tissue revealed by a dual-staining approach. PloS One. (2010) 5:e11108. doi: 10.1371/journal.pone.0011108 20559431 PMC2885421

[52] Basaraba RJ. Experimental tuberculosis: the role of comparative pathology in the discovery of improved tuberculosis treatment strategies. Tuberculosis. (2008) 88:S35–47. doi: 10.1016/s1472-9792(08)70035-0 18762152

[53] McMurray DN. Hematogenous reseeding of the lung in low-dose, aerosol-infected Guinea pigs: unique features of the host-pathogen Interface in secondary tubercles. Tuberculosis (Edinb). (2003) 83:131–4. doi: 10.1016/s1472-9792(02)00079-3 12758202

[54] Kim MJ Wainwright HC Locketz M Bekker LG Walther GB Dittrich C. Caseation of human tuberculosis granulomas correlates with elevated host lipid metabolism. EMBO Mol Med. (2010) 2:258–74. doi: 10.1002/emmm.201000079 20597103 PMC2913288

[55] Pena JC Ho WZ. Non-human primate models of tuberculosis. Microbiol Spectr. (2016) 4. doi: 10.1128/microbiolspec.TBTB2-0007-2016 27726820

[56] Capuano SV Croix DA Pawar S Zinovik A Myers A Lin PL. Experimental *Mycobacterium Tuberculosis* infection of Cynomolgus macaques closely resembles the various manifestations of human *M*. tuberculosis Inf Inf Immun. (2003) 71:5831–44. doi: 10.1128/iai.71.10.5831-5844.2003 PMC201048 14500505

[57] Lin PL Pawar S Myers A Pegu A Fuhrman C Reinhart TA. Early events in *Mycobacterium Tuberculosis* infection in Cynomolgus macaques. Infect Immun. (2006) 74:3790–803. doi: 10.1128/IAI.00064-06 16790751 PMC1489679

[58] Sharpe SA Eschelbach E Basaraba RJ Gleeson F Hall GA McIntyre A. Determination of lesion volume by Mri and stereology in a macaque model of tuberculosis. Tuberculosis (Edinb). (2009) 89:405–16. doi: 10.1016/j.tube.2009.09.002 19879805

[59] Via LE Weiner DM Schimel D Lin PL Dayao E Tankersley SL. Differential virulence and disease progression following *Mycobacterium Tuberculosis* complex infection of the common marmoset (*Callithrix Jacchus*). Infect Immun. (2013) 81:2909–19. doi: 10.1128/IAI.00632-13 23716617 PMC3719573

[60] Sharpe S White A Sarfas C Sibley L Gleeson F McIntyre A. Alternative Bcg delivery strategies improve protection against *Mycobacterium Tuberculosis* in non-human Primates: protection associated with mycobacterial antigen-specific Cd4 effector memory T-cell populations. Tuberculosis. (2016) 101:174–90. doi: 10.1016/j.tube.2016.09.004 27865390 PMC5120991

[61] Walsh GP Tan EV Dela Cruz EC Abalos RM Villahermosa LG Young LJ. The Philippine Cynomolgus monkey (Macaca Fasicularis) provides a New nonhuman primate model of tuberculosis that resembles human disease. Nat Med. (1996) 2:430–6. 8597953 10.1038/nm0496-430

[62] Lin PL Rodgers M Smith L Bigbee M Myers A Bigbee C. Quantitative comparison of active and latent tuberculosis in the Cynomolgus macaque model. Infect Immun. (2009) 77:4631–42. doi: 10.1128/IAI.00592-09 19620341 PMC2747916

[63] Sibley L Dennis M Sarfas C White A Clark S Gleeson F. Route of delivery to the airway influences the distribution of pulmonary disease but not the outcome of *Mycobacterium Tuberculosis* infection in Rhesus macaques. Tuberculosis (Edinb). (2016) 96:141–9. doi: 10.1016/j.tube.2015.11.004 26723465

[64] Cepeda M Salas M Folwarczny J Leandro AC Hodara VL de la Garza MA. Establishment of a neonatal Rhesus macaque model to study *Mycobacterium Tuberculosis* infection. Tuberculosis (Edinb). (2013) 93:S51–9. doi: 10.1016/S1472-9792(13)70011-8 24388650 PMC4051704

[65] Rayner EL Pearson GR Hall GA Basaraba RJ Gleeson F McIntyre A. Early lesions following aerosol infection of Rhesus macaques (*Macaca Mulatta*) with *Mycobacterium Tuberculosis* strain H37rv. J Comp Pathol. (2013) 149:475–85. doi: 10.1016/j.jcpa.2013.05.005 23880551

[66] Rayner EL Pearson GR Hall GA Gleeson F McIntyre A Smyth D. Early lesions following aerosol challenge of Rhesus macaques (*Macaca Mulatta*) with *Mycobacterium Tuberculosis* (Erdman strain). J Comp Pathol. (2015) 152:217–26. doi: 10.1016/j.jcpa.2014.10.002 25481611

[67] Queval CJ Fearns A Botella L Smyth A Schnettger L Mitermite M. Macrophage-specific responses to human- and animal-adapted tubercle Bacilli reveal pathogen and host factors driving multinucleated cell formation. PLoS Pathog. (2021) 17:e1009410. doi: 10.1371/journal.ppat.1009410 33720986 PMC7993774

[68] Hunter L Hingley-Wilson S Stewart GR Sharpe SA Salguero FJ. Dynamics of macrophage, T and B cell infiltration within pulmonary granulomas induced by *Mycobacterium Tuberculosis* in two non-human primate models of aerosol infection. Front Immunol. (2022) 12:776913. doi: 10.3389/fimmu.2021.776913 35069548 PMC8770544

[69] Phuah JY Mattila JT Lin PL Flynn JL. Activated B cells in the granulomas of nonhuman Primates infected with *Mycobacterium Tuberculosis*. Am J Pathol. (2012) 181:508–14. doi: 10.1016/j.ajpath.2012.05.009 22721647 PMC3409439

[70] Aranday-Cortes E Bull NC Villarreal-Ramos B Gough J Hicks D Ortiz-Pelaez A. Upregulation of Il-17a, Cxcl9 and Cxcl10 in early-stage granulomas induced by *Mycobacterium Bovis* in cattle. Transbound Emerg Dis. (2013) 60:525–37. doi: 10.1111/j.1865-1682.2012.01370.x 22909117

[71] Fuller CL Flynn JL Reinhart TA. In situ study of abundant expression of Proinflammatory chemokines and cytokines in pulmonary granulomas that develop in Cynomolgus macaques experimentally infected with *Mycobacterium Tuberculosis*. Infect Immun. (2003) 71:7023–34. doi: 10.1128/IAI.71.12.7023-7034.2003 14638792 PMC308896

[72] Williams A James BW Bacon J Hatch KA Hatch GJ Hall GA. An assay to compare the infectivity of *Mycobacterium Tuberculosis* isolates based on aerosol infection of Guinea pigs and assessment of bacteriology. Tuberculosis (Edinb). (2005) 85:177–84. doi: 10.1016/j.tube.2004.11.001 15850755

[73] Palanisamy GS Smith EE Shanley CA Ordway DJ Orme IM Basaraba RJ. Disseminated disease severity as a measure of virulence of *Mycobacterium Tuberculosis* in the Guinea pig model. Tuberculosis (Edinb). (2008) 88:295–306. doi: 10.1016/j.tube.2007.12.003 18321783 PMC2572689

[74] Larenas-Munoz F Ruedas-Torres I Hunter L Bird A Agullo-Ros I Winsbury R. Characterisation and development of histopathological lesions in a Guinea pig model of *Mycobacterium Tuberculosis* infection. Front Vet Sci. (2023) 10:4200. doi: 10.3389/fvets.2023.1264200 PMC10556493 37808110

[75] Ordway D Palanisamy G Henao-Tamayo M Smith EE Shanley C Orme IM. The cellular immune response to *Mycobacterium Tuberculosis* infection in the Guinea pig. J Immunol. (2007) 179:2532–41. doi: 10.4049/jimmunol.179.4.2532 17675515

[76] Palmer MV Thacker TC Kanipe C Boggiatto PM. Heterogeneity of pulmonary granulomas in cattle experimentally infected with *Mycobacterium Bovis*. Front Vet Sci. (2021) 8:671460. doi: 10.3389/fvets.2021.671460 34026898 PMC8138452

[77] Figl J Köhler H Wedlich N Liebler-Tenorio EM Grode L Parzmair G. Safety and immunogenicity of recombinant Bacille Calmette-Guérin strain Vpm1002 and its derivatives in a goat model. Int J Mol Sci. (2023) 24. doi: 10.3390/ijms24065509 PMC10058566 36982586

[78] Sanchez J Tomás L Ortega N Buendía AJ del Rio L Salinas J. Microscopical and immunological features of Tuberculoid granulomata and Cavitary pulmonary tuberculosis in naturally infected goats. J Comp Pathol. (2011) 145:107–17. doi: 10.1016/j.jcpa.2010.12.006 21334000

[79] Dorman SE Hatem CL Tyagi S Aird K Lopez-Molina J Pitt ML. Susceptibility to tuberculosis: clues from studies with inbred and outbred New Zealand White rabbits. Infect Immun. (2004) 72:1700–5. doi: 10.1128/iai.72.3.1700-1705.2004 14977978 PMC356026

[80] Manabe YC Dannenberg AM Tyagi SK Hatem CL Yoder M Woolwine SC. Different strains of *Mycobacterium Tuberculosis* cause various spectrums of disease in the rabbit model of tuberculosis. Infect Immun. (2003) 71:6004–11. doi: 10.1128/iai.71.10.6004-6011.2003 14500521 PMC201108

[81] Lewinsohn DM Tydeman IS Frieder M Grotzke JE Lines RA Ahmed S. High resolution radiographic and fine immunologic definition of Tb disease progression in the Rhesus macaque. Microbes Infect. (2006) 8:2587–98. doi: 10.1016/j.micinf.2006.07.007 16952476

[82] Flynn JL Klein E. Pulmonary tuberculosis in monkeys In: Leong FJ Dartois V Dick T, editors. A color atlas of comparative pathology of pulmonary tuberculosis. Florida: CRC Press (2011). 83–106.

[83] White AD Sibley L Sarfas C Morrison A Gullick J Clark S. Mtbvac vaccination protects Rhesus macaques against aerosol challenge with M. tuberculosis and induces immune signatures analogous to those observed in clinical studies. NPJ Vaccines. (2021) 6:4. doi: 10.1038/s41541-020-00262-8 33397991 PMC7782851

[84] Sugawara I Yamada H Mizuno S. Nude Rat (F344/N-Rnu) Tuberculosis. Cell Microbiol. (2006) 8:661–7. doi: 10.1111/j.1462-5822.2005.00658.x 16548891

[85] Sugawara I Yamada H Mizuno S. Pathological and immunological profiles of rat tuberculosis. Int J Exp Pathol. (2004) 85:125–34. doi: 10.1111/j.0959-9673.2004.00379.x 15255966 PMC2517471

[86] Blanco JC Boukhvalova MS Perez DR Vogel SN Kajon A. Modeling human respiratory viral infections in the cotton rat (*Sigmodon Hispidus*). J Antivir Antiretrovir. (2014) 6:40–2. doi: 10.4172/jaa.1000093 25635205 PMC4307615

[87] Clark S Hall Y Williams A. Animal models of tuberculosis: Guinea pigs. Cold Spring Harb Perspect Med. (2014) 5:a018572. doi: 10.1101/cshperspect.a018572 25524720 PMC4448592

[88] Flynn JL. Lessons from experimental *Mycobacterium Tuberculosis* infections. Microbes Infect. (2006) 8:1179–88. doi: 10.1016/j.micinf.2005.10.033 16513383

[89] Kaplan G Tsenova L. Pulmonary tuberculosis in the rabbit In: Leong FJDV Dick T, editors. A color atlas of comparative pathology of pulmonary tuberculosis Boca Raton. Florida: CRC Press (2011). 107–30.

[90] Tsenova L Sokol K Freedman VH Kaplan G. A combination of thalidomide plus antibiotics protects rabbits from mycobacterial meningitis-associated death. J Infect Dis. (1998) 177:1563–72. doi: 10.1086/515327 9607834

[91] Jassal MS Nedeltchev GG Osborne J Bishai WR. A modified scoring system to describe gross pathology in the rabbit model of tuberculosis. BMC Microbiol. (2011) 11:49. doi: 10.1186/1471-2180-11-49 21375756 PMC3058006

[92] Lurie MB. The fate of tubercle Bacilli in the organs of Reinfected rabbits. J Exp Med. (1929) 50:747–65. doi: 10.1084/jem.50.6.747 19869661 PMC2131660

[93] Lurie MB. The correlation between the histological changes and the fate of living tubercle Bacilli in the organs of tuberculous rabbits. J Exp Med. (1932) 55:31–54. doi: 10.1084/jem.55.1.31 19869977 PMC2132067

[94] Subbian S Tsenova L Yang G O'Brien P Parsons S Peixoto B. Chronic pulmonary Cavitary tuberculosis in rabbits: a failed host immune response. Open Biol. (2011) 1:110016. doi: 10.1098/rsob.110016 22645653 PMC3352086

[95] Salguero FJ. The pathology and pathogenesis of Mycobacterium bovis infection. CABI Books. CABI. (2018):122–39. doi: 10.1079/9781786391520.0122

[96] García-Jiménez WL Benítez-Medina JM Fernández-Llario P Abecia JA García-Sánchez A Martínez R. Comparative pathology of the natural infections by Mycobacterium Bovis and by *Mycobacterium Caprae* in wild boar (*Sus Scrofa*). Transbound Emerg Dis. (2013) 60:102–9. doi: 10.1111/j.1865-1682.2012.01321.x 22469036

[97] Palmer MV Wiarda J Kanipe C Thacker TC. Early pulmonary lesions in cattle infected Via aerosolized *Mycobacterium Bovis*. Vet Pathol. (2019) 56:544–54. doi: 10.1177/0300985819833454, PMID: 30895908

[98] Palmer MV Waters WR Thacker TC. Lesion development and Immunohistochemical changes in granulomas from cattle experimentally infected with *Mycobacterium Bovis*. Vet Pathol. (2007) 44:863–74. doi: 10.1354/vp.44-6-863 18039899

[99] Kanipe C Boggiatto PM Putz EJ Palmer MV. Histopathologic differences in granulomas of *Mycobacterium Bovis* Bacille Calmette Guerin (Bcg) vaccinated and non-vaccinated cattle with bovine tuberculosis. Front Microbiol. (2022) 13:1048648. doi: 10.3389/fmicb.2022.1048648 36425039 PMC9678917

[100] Palmer MV Thacker TC Waters WR. Multinucleated Giant cell cytokine expression in pulmonary granulomas of cattle experimentally infected with *Mycobacterium Bovis*. Vet Immunol Immunopathol. (2016) 180:34–9. doi: 10.1016/j.vetimm.2016.08.015 27692093

[101] Menin A Fleith R Reck C Marlow M Fernandes P Pilati C. Asymptomatic cattle naturally infected with *Mycobacterium Bovis* present exacerbated tissue pathology and bacterial dissemination. PloS One. (2013) 8:e53884. doi: 10.1371/journal.pone.0053884 23326525 PMC3541226

[102] Johnson LK Liebana E Nunez A Spencer Y Clifton-Hadley R Jahans K. Histological observations of bovine tuberculosis in lung and lymph node tissues from British deer. Vet J. (2008) 175:409–12. doi: 10.1016/j.tvjl.2007.04.021 17584504

[103] Cassidy JP Bryson DG Gutiérrez Cancela MM Forster F Pollock JM Neill SD. Lymphocyte subtypes in experimentally induced early-stage bovine tuberculous lesions. J Comp Pathol. (2001) 124:46–51. doi: 10.1053/jcpa.2000.0427 11428188

[104] Cassidy JP Bryson DG Pollock JM Evans RT Forster F Neill SD. Early lesion formation in cattle experimentally infected with *Mycobacterium Bovis*. J Comp Pathol. (1998) 119:27–44. doi: 10.1016/S0021-9975(98)80069-8 9717125

[105] Gonzalez-Juarrero M Bosco-Lauth A Podell B Soffler C Brooks E Izzo A. Experimental aerosol *Mycobacterium Bovis* model of infection in goats. Tuberculosis (Edinb). (2013) 93:558–64. doi: 10.1016/j.tube.2013.05.006 23850102

[106] Gong W Liang Y Wu X. Animal models of tuberculosis vaccine research: an important component in the fight against tuberculosis. Biomed Res Int. (2020) 2020:4263079. doi: 10.1155/2020/4263079 32025519 PMC6984742

[107] Wedlich N Figl J Liebler-Tenorio EM Köhler H von Pückler K Rissmann M. Video endoscopy-guided Intrabronchial spray inoculation of *Mycobacterium Bovis* in goats and comparative assessment of lung lesions with various imaging methods. Frontiers in veterinary. Science. (2022):9. doi: 10.3389/fvets.2022.877322 35591868 PMC9113525

[108] de Val PB López-Soria S Nofrarías M Martín M Vordermeier HM Villarreal-Ramos B. Experimental model of tuberculosis in the domestic goat after endobronchial infection with *Mycobacterium Caprae*. Clin Vaccine Immunol. (2011) 18:1872–81. doi: 10.1128/cvi.05323-11 21880849 PMC3209027

[109] Seva J Menchén V Navarro JA Pallarés FJ Villar D Vásquez F. Caprine tuberculosis eradication program: an Immunohistochemical study. Small Rumin Res. (2002) 46:107–14. doi: 10.1016/S0921-4488(02)00174-8

[110] Bezos J de Juan L Romero B Álvarez J Mazzucchelli F Mateos A. Experimental infection with *Mycobacterium Caprae* in goats and evaluation of immunological status in tuberculosis and Paratuberculosis co-infected animals. Vet Immunol Immunopathol. (2010) 133:269–75. doi: 10.1016/j.vetimm.2009.07.018 19716181

[111] Vordermeier HM Chambers MA Cockle PJ Whelan AO Simmons J Hewinson RG. Correlation of Esat-6-specific gamma interferon production with pathology in cattle following *Mycobacterium Bovis* Bcg vaccination against experimental bovine tuberculosis. Infect Immun. (2002) 70:3026–32. doi: 10.1128/iai.70.6.3026-3032.2002 12010994 PMC128013

[112] Roy Á Risalde MA Bezos J Casal C Romero B Sevilla I. Response of goats to intramuscular vaccination with heat-killed Mycobacterium Bovis and natural challenge. Comp Immunol Microbiol Infect Dis. (2018) 60:28–34. doi: 10.1016/j.cimid.2018.09.006 30396427

[113] Roy A Tomé I Romero B Lorente-Leal V Infantes-Lorenzo JA Domínguez M. Evaluation of the immunogenicity and efficacy of Bcg and Mtbvac vaccines using a natural transmission model of tuberculosis. Vet Res. (2019) 50:82. doi: 10.1186/s13567-019-0702-7 31615555 PMC6792192

[114] Domingo M Vidal E Marco A. Pathology of bovine tuberculosis. Res Vet Sci. (2014) 97:S20–9. doi: 10.1016/j.rvsc.2014.03.017 24731532

[115] Neila C Rebollada-Merino A Bezos J de Juan L Domínguez L Rodríguez-Bertos A. Extracellular matrix proteins (fibronectin, collagen iii, and collagen I) Immunoexpression in goat tuberculous granulomas (*Mycobacterium Caprae*). Vet Res Commun. (2022) 46:1147–56. doi: 10.1007/s11259-022-09996-3 36136210 PMC9684263

[116] Carrisoza-Urbina J Morales-Salinas E Bedolla-Alva MA Hernández-Pando R Gutiérrez-Pabello JA. Atypical granuloma formation in *Mycobacterium Bovis*-infected calves. PloS One. (2019) 14:e0218547. doi: 10.1371/journal.pone.0218547 31306432 PMC6629060

[117] di Marco Lo Presti V Capucchio MT Fiasconaro M Puleio R la Mancusa F Romeo G. *Mycobacterium Bovis* tuberculosis in two goat farms in multi-host ecosystems in Sicily (Italy): epidemiological, diagnostic, and regulatory considerations. Pathogens. (2022) 11. doi: 10.3390/pathogens11060649, PMID: 35745503 PMC9230833

[118] Liebler-Tenorio EM Heyl J Wedlich N Figl J Kohler H Krishnamoorthy G. Vaccine-induced subcutaneous granulomas in goats reflect differences in host-Mycobacterium interactions between Bcg- and recombinant Bcg-derivative vaccines. Int J Mol Sci. (2022) 23. doi: 10.3390/ijms231910992 PMC9570401 36232295

[119] Balseiro A Altuzarra R Vidal E Moll X Espada Y Sevilla IA. Assessment of Bcg and inactivated *Mycobacterium Bovis* vaccines in an experimental tuberculosis infection model in sheep. PloS One. (2017) 12:e0180546. doi: 10.1371/journal.pone.0180546 28678885 PMC5498051

[120] Garcia-Jimenez WL Fernandez-Llario P Gomez L Benitez-Medina JM Garcia-Sanchez A Martinez R. Histological and Immunohistochemical characterisation of *Mycobacterium Bovis* induced granulomas in naturally infected fallow deer (*Dama Dama*). Vet Immunol Immunopathol. (2012) 149:66–75. doi: 10.1016/j.vetimm.2012.06.010 22763148

[121] Palmer MV Waters WR Whipple DL. Lesion development in White-tailed deer (*Odocoileus Virginianus*) experimentally infected with *Mycobacterium Bovis*. Vet Pathol. (2002) 39:334–40. doi: 10.1354/vp.39-3-334 12014497

[122] Bouz G Al HN. The zebrafish model of tuberculosis - no lungs needed. Crit Rev Microbiol. (2018) 44:779–92. doi: 10.1080/1040841X.2018.1523132 30663918

[123] Ramakrishnan L. Looking within the zebrafish to understand the tuberculous granuloma. Adv Exp Med Biol. (2013) 783:251–66. doi: 10.1007/978-1-4614-6111-1_13 23468113

[124] Meijer AH. Protection and pathology in Tb: learning from the zebrafish model. Semin Immunopathol. (2016) 38:261–73. doi: 10.1007/s00281-015-0522-4 26324465 PMC4779130

[125] Myllymaki H Bauerlein CA Ramet M. The zebrafish breathes New life into the study of tuberculosis. Front Immunol. (2016) 7:196. doi: 10.3389/fimmu.2016.00196 27242801 PMC4871865

[126] Zon LI Peterson RT. In vivo drug discovery in the zebrafish. Nat Rev Drug Discov. (2005) 4:35–44. doi: 10.1038/nrd1606 15688071

[127] Varela M Meijer AH. A fresh look at mycobacterial pathogenicity with the zebrafish host model. Mol Microbiol. (2022) 117:661–9. doi: 10.1111/mmi.14838 34714579 PMC9297993

[128] Prouty MG Correa NE Barker LP Jagadeeswaran P Klose KE. Zebrafish-*Mycobacterium Marinum* model for mycobacterial pathogenesis. FEMS Microbiol Lett. (2003) 225:177–82. doi: 10.1016/S0378-1097(03)00446-4 12951238

[129] Swaim LE Connolly LE Volkman HE Humbert O Born DE Ramakrishnan L. *Mycobacterium Marinum* infection of adult zebrafish causes Caseating granulomatous tuberculosis and is moderated by adaptive immunity. Infect Immun. (2006) 74:6108–17. doi: 10.1128/IAI.00887-06 17057088 PMC1695491

[130] Davis JM Clay H Lewis JL Ghori N Herbomel P Ramakrishnan L. Real-time visualization of Mycobacterium-macrophage interactions leading to initiation of granuloma formation in zebrafish embryos. Immunity. (2002) 17:693–702. doi: 10.1016/s1074-7613(02)00475-2 12479816

[131] van Leeuwen LM van der Sar AM Bitter W. Animal models of tuberculosis: zebrafish. Cold Spring Harb Perspect Med. (2014) 5:a018580. doi: 10.1101/cshperspect.a018580 25414379 PMC4355259

[132] Parikka M Hammaren MM Harjula SK Halfpenny NJ Oksanen KE Lahtinen MJ. *Mycobacterium Marinum* causes a latent infection that can be reactivated by gamma irradiation in adult zebrafish. PLoS Pathog. (2012) 8:e1002944. doi: 10.1371/journal.ppat.1002944 23028333 PMC3459992

[133] Yoon S Alnabulsi A Wang TY Lee PT Chen T-Y Bird S. Analysis of interferon gamma protein expression in zebrafish (*Danio Rerio*). Fish Shellfish Immunol. (2016) 57:79–86. doi: 10.1016/j.fsi.2016.08.023 27539703

[134] Saralahti AK Uusi-Makela MIE Niskanen MT Ramet M. Integrating fish models in tuberculosis vaccine development. Dis Model Mech. (2020) 13. doi: 10.1242/dmm.045716 PMC7473647 32859577

[135] Risalde MA López V Contreras M Mateos-Hernández L Gortázar C de la Fuente J. Control of Mycobacteriosis in zebrafish (*Danio Rerio*) Mucosally vaccinated with heat-inactivated *Mycobacterium Bovis*. Vaccine. (2018) 36:4447–53. doi: 10.1016/j.vaccine.2018.06.042 29935860

[136] Tobin DM Ramakrishnan L. Comparative pathogenesis of Mycobacterium Marinum and *Mycobacterium Tuberculosis*. Cell Microbiol. (2008) 10:1027–39. doi: 10.1111/j.1462-5822.2008.01133.x 18298637

[137] Stoop EJ Schipper T Rosendahl Huber SK Nezhinsky AE Verbeek FJ Gurcha SS. Zebrafish embryo screen for mycobacterial genes involved in the initiation of granuloma formation reveals a newly identified Esx-1 component. Dis Model Mech. (2011) 4:526–36. doi: 10.1242/dmm.006676 21372049 PMC3124061

[138] López V Risalde MA Contreras M Mateos-Hernández L Vicente J Gortázar C. Heat-inactivated *Mycobacterium Bovis* protects zebrafish against Mycobacteriosis. J Fish Dis. (2018) 41:1515–28. doi: 10.1111/jfd.12847 29956837

[139] Fitzgerald SD Kaneene JB. Wildlife reservoirs of bovine tuberculosis worldwide: hosts, pathology, surveillance, and control. Vet Pathol. (2013) 50:488–99. doi: 10.1177/0300985812467472 23169912

[140] Garcia-Jimenez WL Salguero FJ Fernandez-Llario P Martinez R Risco D Gough J. Immunopathology of granulomas produced by *Mycobacterium Bovis* in naturally infected wild boar. Vet Immunol Immunopathol. (2013) 156:54–63. doi: 10.1016/j.vetimm.2013.09.008 24144683

[141] Risco D Salguero FJ Cerrato R Gutierrez-Merino J Lanham-New S Barquero-Pérez O. Association between vitamin D supplementation and severity of tuberculosis in wild boar and Red Deer. Res Vet Sci. (2016) 108:116–9. doi: 10.1016/j.rvsc.2016.08.003 27663379

[142] Gil O Diaz I Vilaplana C Tapia G Diaz J Fort M. Granuloma encapsulation is a key factor for containing tuberculosis infection in Minipigs. PloS One. (2010) 5:e10030. doi: 10.1371/journal.pone.0010030 20386605 PMC2850319

[143] Chambers MA Aldwell F Williams GA Palmer S Gowtage S Ashford R. The effect of Oral vaccination with *Mycobacterium Bovis* Bcg on the development of tuberculosis in captive European badgers (*Meles Meles*). Front Cell Infect Microbiol. (2017) 7:6. doi: 10.3389/fcimb.2017.00006 28174695 PMC5258709

[144] Corner LA Murphy D Gormley E. *Mycobacterium Bovis* infection in the Eurasian badger (*Meles Meles*): the disease, pathogenesis. Epidemiol Control J Comp Pathol. (2011) 144:1–24. doi: 10.1016/j.jcpa.2010.10.003 21131004

[145] Lesellier S Palmer S Gowtage-Sequiera S Ashford R Dalley D Davé D. Protection of Eurasian badgers (*Meles Meles*) from tuberculosis after intra-muscular vaccination with different doses of Bcg. Vaccine. (2011) 29:3782–90. doi: 10.1016/j.vaccine.2011.03.028 21440035

[146] Balseiro A Prieto JM Álvarez V Lesellier S Davé D Salguero FJ. Protective effect of Oral Bcg and inactivated *Mycobacterium Bovis* vaccines in European badgers (*Meles Meles*) experimentally infected with *M*. *bovis* Front Vet Sci. (2020) 7:41. doi: 10.3389/fvets.2020.00041 32118064 PMC7011093

[147] Senior NJ Bagnall MC Champion OL Reynolds SE La Ragione RM Woodward MJ. Galleria Mellonella as an infection model for *Campylobacter Jejuni* virulence. J Med Microbiol. (2011) 60:661–9. doi: 10.1099/jmm.0.026658-0 21233296

[148] Li Y Spiropoulos J Cooley W Khara JS Gladstone CA Asai M. Galleria Mellonella - a novel infection model for the *Mycobacterium Tuberculosis* complex. Virulence. (2018) 9:1126–37. doi: 10.1080/21505594.2018.1491255 30067135 PMC6086298

[149] Asai M Li Y Spiropoulos J Cooley W Everest DJ Kendall SL. Galleria Mellonella as an infection model for the virulent *Mycobacterium Tuberculosis* H37rv. Virulence. (2022) 13:1543–57. doi: 10.1080/21505594.2022.2119657 36052440 PMC9481108

[150] Thacker VV Dhar N Sharma K Barrile R Karalis K McKinney JD. A lung-on-Chip model of early *Mycobacterium Tuberculosis* infection reveals an essential role for alveolar epithelial cells in controlling bacterial growth. Elife. (2020) 9:9961. doi: 10.7554/eLife.59961 PMC7735758 33228849

[151] Duffy SC Srinivasan S Schilling MA Stuber T Danchuk SN Michael JS. Reconsidering *Mycobacterium Bovis* as a proxy for zoonotic tuberculosis: a molecular epidemiological surveillance study. Lancet Microbe. (2020) 1:e66–73. doi: 10.1016/S2666-5247(20)30038-0 32642742 PMC7325494

